# Integration of nanobiosensors into organ-on-chip systems for monitoring viral infections

**DOI:** 10.1186/s40580-024-00455-0

**Published:** 2024-11-26

**Authors:** Jiande Zhang, Min-Hyeok Kim, Seulgi Lee, Sungsu Park

**Affiliations:** 1 School of Mechanical Engineering, Sungkyunkwan University (SKKU), Suwon, 16419 Korea; 2 Department of Metabiohealth, Sungkyunkwan University (SKKU), Suwon, 16419 Korea; 3 Department of Biophysics, Institute of Quantum Biophysics (IQB), Sungkyunkwan University (SKKU), Suwon, 16419 Korea

**Keywords:** Organ-on-chip, Nanobiosensors, Viral infections, Monitoring, Integration, Cytokines

## Abstract

The integration of nanobiosensors into organ-on-chip (OoC) models offers a promising advancement in the study of viral infections and therapeutic development. Conventional research methods for studying viral infection, such as two-dimensional cell cultures and animal models, face challenges in replicating the complex and dynamic nature of human tissues. In contrast, OoC systems provide more accurate, physiologically relevant models for investigating viral infections, disease mechanisms, and host responses. Nanobiosensors, with their miniaturized designs and enhanced sensitivity, enable real-time, continuous, in situ monitoring of key biomarkers, such as cytokines and proteins within these systems. This review highlights the need for integrating nanobiosensors into OoC systems to advance virological research and improve therapeutic outcomes. Although there is extensive literature on biosensors for viral infection detection and OoC models for replicating infections, real integration of biosensors into OoCs for continuous monitoring remains unachieved. We discuss the advantages of nanobiosensor integration for real-time tracking of critical biomarkers within OoC models, key biosensor technologies, and current OoC systems relevant to viral infection studies. Additionally, we address the main technical challenges and propose solutions for successful integration. This review aims to guide the development of biosensor-integrated OoCs, paving the way for precise diagnostics and personalized treatments in virological research.

**Graphical Abstract:**

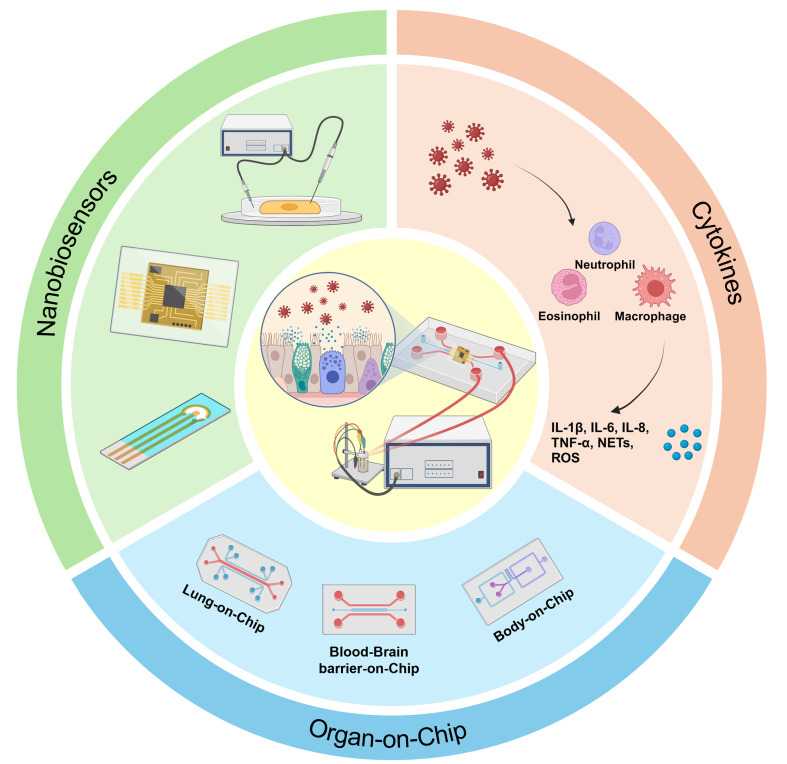

## Introduction

Viral infections significantly contribute to global morbidity and mortality, presenting a major health burden worldwide [1, 2]. Diseases caused by viruses like human immunodeficiency virus, influenza, and respiratory syncytial virus have long been significant public health concerns. The recent outbreak of COVID-19 has led to millions of infections and deaths, with profound and lasting impacts on public health and the global economy. Additionally, new viral threats, such as Mpox [3], continue to emerge, while vaccine development for these diseases often lags due to limited knowledge of the viruses. Therefore, studying viral infection mechanisms is crucial for developing effective prevention strategies and accelerating vaccine development.

In traditional models, such as animal studies and 2D cell cultures, biosensors face limitations due to the inability of these models to fully replicate the complex, dynamic environment of human tissues during viral infection. These limitations hinder the effectiveness of biosensors in capturing real-time infection dynamics and key biomarkers, which are essential for studying viral infections accurately. For example, in 2D cell cultures, the flat growth environment fails to replicate the complex, 3D architecture of human tissues, which is crucial for understanding cellular behavior and immune responses in a realistic context [4]. This lack of structural complexity limits the ability of biosensors to accurately monitor infection dynamics and cellular interactions as they would occur in vivo. Animal models, though commonly used, often yield responses that do not translate to human physiology due to species differences, reducing the relevance of biosensor data for human applications. Additionally, these models lack the precision needed for real-time, in situ monitoring. For instance, animal models often require invasive sampling, while 2D cultures rely on endpoint assays, which interrupt dynamic processes and limit continuous tracking [5]. Traditional models also struggle with limited control over the cellular microenvironment, preventing accurate representation of viral progression and host response [6]. Multiplexing capabilities are often restricted, with biosensors typically detecting only single or limited biomarkers, which fail to capture the complexity of immune responses. These ethical and practical constraints underscore the need for more effective alternatives.

Recent advances in microfluidic organ-on-chip (OoC) systems have led to the development of biomimetic 3D human tissue models. OoC devices are microengineered devices that replicate the microarchitecture and functions of human organs on a small scale, enabling the simulation of complex biological processes in a controlled environment [7–10]. Compared to traditional 2D cultures and animal models, OoCs provide more reliable and accurate experimental outcomes, offering a closer approximation to the in vivo human environment [11, 12]. Longitudinal detection of viral biomarkers in OoC models is particularly crucial for monitoring viral infections to understand their progression and potential treatment. Real-time monitoring provides invaluable insights into the dynamics of viral infections and the efficacy of antiviral treatments, underscoring the importance of integrating nanobiosensors into OoC devices.

Despite the advantages of OoCs, integrating biosensors into these platforms presents challenges due to the size of traditional biosensors, which are often too large to fit seamlessly into the microengineered devices [13–15]. Nanobiosensors, with their significantly smaller size and increased sensitivity, provide a practical solution to this issue. By miniaturizing the sensing technology, nanobiosensors can be effectively incorporated into OoC models, allowing real-time, continuous, and in situ monitoring of physiological and pathological changes.

**Fig. 1:**
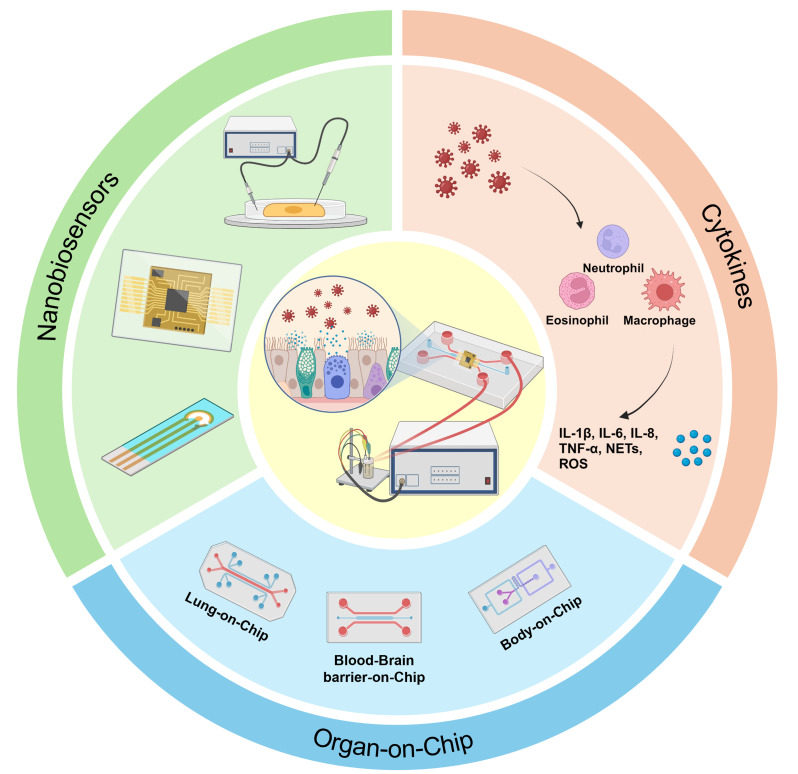
Nanobiosensor and immune cell-integrated Organ-on-Chip (OoC) models for virus infection monitoring. Nanobiosensors provide continuous, high-sensitivity tracking of biomarkers in real time. Cytokines indicate immune responses to infection, including key inflammatory markers, such as IL-1β, IL-6, and TNF-α. OoC models, such as lung, blood-brain barrier, and multi-organ models, simulate various organ environments to study the dynamics of viral infections in a controlled setting. These components work together to enable real-time monitoring of immune responses within organ-on-chip. (Created with Biorender.com)

This review aims to address the critical need for integrating biosensors into OoC systems for viral infection monitoring, a field that, to date, lacks practical examples of such integration. Although OoC systems and biosensors have been extensively studied independently for their applications in virology, there has been little progress in combining these technologies to create a comprehensive, real-time monitoring platform for viral infections. By reviewing existing biosensor technologies and OoC models, we aim to outline a path forward for researchers seeking to incorporate biosensors into OoC systems, enabling continuous, in situ tracking of viral dynamics within a biomimetic environment. Specifically, this review highlights the benefits and potential strategies for integrating nanobiosensors into OoCs, focusing on key biomarkers for effective viral infection monitoring (Fig. 1). We also discuss the primary technical challenges involved in this integration and propose possible solutions to overcome these obstacles. Our hope is that this review will guide future research efforts, providing a foundation for developing advanced OoC platforms that support more accurate virological research and improved therapeutic strategies.

## Biomarkers and biosensors for monitoring viral infection

To monitor viral infections, cytokines play a crucial role as key signaling molecules in the immune response, with their levels varying significantly, offering insights into the infection’s severity and immune activity. They are typically classified into proinflammatory cytokines, anti-inflammatory cytokines, and chemokines. Additionally, other noncytokine biomarkers, such as viral antigen, viral load, and C-reactive protein, can also provide valuable information about the infection and its progression [16, 17]. The following section will discuss these cytokines and noncytokine biomarkers in detail, elaborating on their specific roles in viral infections and their significance in disease monitoring.

### Cytokines and cytokine storms

Cytokines are essential signaling molecules in the immune response, and their levels can fluctuate significantly during viral infections, offering insights into both the severity of the infection and the immune response. Their specific roles in viral infections and their importance in disease monitoring will be discussed in further detail.

Interleukin-6 (IL-6) is a key proinflammatory cytokine involved in the acute phase response, immune regulation, and hematopoiesis. It stimulates the production of acute-phase proteins like C-reactive protein (CRP) and fibrinogen [18, 19]. In healthy individuals, the average IL-6 level is around 5.2 pg/mL [20]. However, during viral infections such as COVID-19, IL-6 levels can rise dramatically, sometimes exceeding 100 pg/mL, especially in severe cases [21].

Interleukin-8, also known as CXCL8, is a chemokine that recruits and activates neutrophils, triggering the release of inflammatory mediators. Elevated IL-8 levels have been observed in severe cases of COVID-19, highlighting its role in the disease’s progression [22, 23].

Tumor Necrosis Factor-alpha (TNF-α) is a key proinflammatory cytokine involved in systemic inflammation, playing a role in inducing fever, shock, tissue injury, and, in severe cases, sepsis [24]. In healthy individuals, TNF-α levels are typically around 5.5 pg/mL [25]. However, during severe viral infections, such as those caused by Ebola or influenza, TNF-α levels can rise significantly, often exceeding 100 pg/mL [26, 27]. Elevated TNF-α levels are commonly associated with severe inflammation and tissue damage [28].

Interleukin-1 beta (IL-1β) is another potent proinflammatory cytokine that induces fever and contributes to the inflammatory response by promoting the release of other cytokines [29]. Under normal conditions, IL-1β levels are generally low, ranging from 0.14 to 1 pg/mL in healthy individuals [30]. However, during viral infections, particularly those with systemic effects like COVID-19, IL-1β levels can rise to 7.9–19.5 pg/mL [31]. High IL-1β levels are associated with severe systemic inflammation and may play a role in the development of cytokine storms [32].

Interferon-gamma (IFN-γ) is a crucial cytokine in antiviral immunity, known for stimulating macrophages and enhancing antigen presentation. It also exhibits direct antiviral effects by inhibiting viral replication. In healthy individuals, IFN-γ levels are generally undetectable or very low, typically below 20 pg/mL [33]. However, during viral infections such as influenza or hepatitis, IFN-γ levels can rise to 20–100 pg/mL, depending on the severity of the infection [33, 34]. Extremely high IFN-γ levels may indicate a hyperactive immune response, contributing to immunopathology.

Cytokine storms represent an extreme immune response in which the body releases an overwhelming amount of proinflammatory cytokines in reaction to an infection. This hyperactive response is particularly dangerous because it can cause severe tissue damage, multiorgan failure, and death. During a cytokine storm, several key cytokines become significantly elevated. IL-6 levels, for example, can exceed 80 pg/mL, serving as a critical indicator of the storm’s severity and a strong predictor of poor outcomes [35]. Similarly, TNF-α levels can rise dramatically, often reaching or surpassing 100 pg/mL [26, 27], playing a central role in systemic inflammation and contributing to the tissue damage observed during cytokine storms [24]. Moreover, in cytokine storms, characterized by the uncontrolled release of proinflammatory cytokines, IL-10 often becomes elevated as the body attempts to counterbalance the excessive inflammation caused by other cytokines like IL-1β, TNF-α, and IL-6 [36]. These elevated cytokine levels drive the pathological process of cytokine storms, leading to severe clinical manifestations and underscoring the importance of early and effective intervention. Monitoring these cytokine dynamics in OoC systems is essential for understanding and mitigating complications associated with cytokine storms.

### Other biomarkers

In addition to cytokines, traditional methods also monitor various other biomarkers to provide a comprehensive view of viral infections. Viral antigens are commonly detected using techniques such as lateral flow assays and ELISA, while the genes encoding these antigens are identified using quantitative PCR (qPCR). However, these biosensing tools, due to their assay formats, may not be easily integrated into OoC systems [37]. For a detailed review of these viral antigens and their detection methods, readers can refer to specialized literature on the subject [38].

C-reactive protein (CRP) is an acute-phase protein that increases in response to inflammation. During viral infections, CRP levels can rise significantly, often reaching 10–60 mg/L [39]. In severe cases, such as COVID-19, levels may exceed 100 mg/L [40]. Elevated CRP levels indicate systemic inflammation, which is common in viral infections. Although CRP is nonspecific, it is widely used to monitor the overall inflammatory state.

Ferritin, a blood protein that stores iron, is commonly elevated in conditions involving inflammation or excessive iron storage [41]. Normal Ferritin levels range from 9 to 136 µg/L in women and 35–220 µg/L in men [42]. However, during severe viral infections, particularly those involving hyperinflammatory responses like COVID-19, Ferritin levels can spike dramatically, often reaching 254.1 ± 33.73 in women and 525.6 ± 69.55 µg/L in men [43]. These elevated levels are particularly significant in diagnosing and managing hyperinflammatory states, including cytokine storms.

Viral load is a critical marker for monitoring viral infections. qPCR measures the amount of viral RNA or DNA in a patient’s body fluids, providing direct evidence of the virus’s presence and quantity. For instance, in SARS-CoV-2 infections, viral load can vary considerably depending on the infection stage, with higher loads typically observed during the early and symptomatic phases. A viral load exceeding 10^10^ copies/mL is often considered high and is associated with increased transmissibility and severity [44]. Monitoring viral load is essential for assessing the infection’s stage and severity.

Liver enzymes, such as alanine aminotransferase (ALT) and aspartate aminotransferase (AST), are important to monitor because viral infections, particularly those affecting the liver (e.g., hepatitis) or systemic infections like COVID-19, can lead to liver damage [45]. Normal ALT levels range from 30 to 65 units per liter (U/L), and AST levels range from 15 to 37 U/L [46]. During viral infections, especially hepatitis, ALT and AST levels can rise significantly, with median values found to be 127.7 and 95.0 U/L, indicating liver involvement and damage [47].

Lactate dehydrogenase (LDH) is an enzyme present in nearly all living cells and is released during tissue damage [48]. Normal LDH levels for adults typically range from 122 to 222 U/L (as indicated by Mayo Clinic Labs). However, in severe viral infections such as COVID-19, LDH levels can rise significantly, often exceeding 1,000 U/L, reflecting extensive tissue injury [49]. Elevated LDH levels are commonly used to assess the extent of tissue damage and can serve as a marker of disease severity.

White blood cell (WBC) counts, including lymphocytes, neutrophils, and other subtypes, provide insights into the immune system’s response to viral infection. Normal WBC counts range from 4 to 11 billion cells per liter of blood [50]. During viral infections, a decrease in lymphocytes (lymphopenia) is frequently observed, particularly in severe cases like COVID-19, where lymphocyte counts can fall below 1,000 cells/µL, indicating a suppressed immune response [51].

D-dimer is a fibrin degradation product used primarily to assess clot formation and breakdown [52]. Normal D-dimer levels are generally below 0.5 µg/mL. Elevated D-dimer levels, often exceeding 1.0 µg/mL, suggest increased clotting activity and are commonly observed in severe viral infections such as COVID-19, where levels can rise significantly, sometimes surpassing 1.8 µg/mL [53]. Monitoring D-dimer is important for evaluating the risk of thrombotic complications, which are prevalent in severe cases [54].

Antibody titers, including specific antibodies such as IgM and IgG against the virus, help determine the stage of infection (acute vs. convalescent) and the patient’s immune response [55]. For instance, IgM antibodies typically appear early in the infection, detectable within the first week and peaking around 1–2 weeks. IgG antibodies develop later, indicating past infection or longer-term immunity, with levels varying depending on the pathogen [56].

## Biosensor for detecting biomarkers in virus infection cases

### Conventional biosensors

Traditional methods for monitoring viral infections, such as molecular techniques like PCR and ELISA, have been invaluable for detecting viral particles and biomarkers. However, these methods often fall short in real-time, continuous monitoring, which is essential for understanding the dynamic nature of viral infections. Most traditional techniques are endpoint assays that require multiple steps and provide intermittent snapshots of biomarker levels, rather than continuous data. Additionally, these methods can be time-consuming, invasive, and unable to capture rapid changes in cytokine release, tissue integrity, or immune responses.

Biosensors address these limitations by offering real-time, continuous monitoring of key biomarkers such as cytokines, proteins, and antibodies in a noninvasive and highly sensitive manner. Integrating these biosensors into OoC systems allows researchers to track the progression of viral infections and the body’s response over time, yielding more detailed insights into disease mechanisms. Unlike traditional methods, biosensors provide instant feedback and can be miniaturized for integration with complex microfluidic systems, making them particularly well-suited for applications in OoC models.

Electrochemical, optical, piezoelectric, and thermal biosensors are particularly valuable in these applications because of their ability to detect biomarker fluctuations with high specificity and sensitivity. Each technology offers distinct advantages: electrochemical biosensors measure electrical signals generated by biomolecular interactions, optical biosensors track changes in light properties, piezoelectric biosensors detect mass changes, and thermal biosensors detect monitor temperature shifts associated with biomarker interactions.

The integration of biosensors into OoC systems represents a significant advancement over traditional methods. It enables dynamic, real-time monitoring of viral infections and provides continuous, in situ data. This shift from static, endpoint assays to dynamic, integrated systems is a critical step forward in understanding and treating viral diseases.

Electrochemical biosensors detect electrical signals generated from the interaction between a target biomarker and the biosensor’s recognition element. These biosensors are widely used due to their high sensitivity, selectivity, and potential for miniaturization. For example, in the context of SARS-CoV-2 infection, electrochemical biosensors have been employed to detect CRP, a marker for inflammation. These biosensors can achieve a limit of detection (LOD) for CRP as low as 0.1 ng/mL. Studies have demonstrated that electrochemical biosensors were effectively monitor CRP levels, providing a sensitive and rapid method for assessing inflammation during viral infection [57]. Similarly, electrochemical biosensors have been used to monitor alanine aminotransferase (ALT), a liver enzyme, in Hepatitis B Virus (HBV) infection. These biosensors can detect ALT with an LOD of 5 U/L, making them valuable for assessing liver damage in HBV patients [58].

Optical biosensors detect changes in optical properties such as absorbance, fluorescence, luminescence, or surface plasmon resonance (SPR) when the target biomarker interacts with the biosensor. These biosensors are especially useful for real-time, label-free detection and are highly sensitive. For instance, in monitoring of SARS-CoV-2, SPR biosensors have been used to detect antibodies (IgM and IgG) against the virus, with an LOD as low as 12.75 ng/mL, providing crucial information about the immune response during infection [59]. SPR biosensors have also been utilized to detect Ferritin, a marker of hyperinflammation, with an LOD of 12 ng/mL, which is particularly relevant for severe COVID-19 cases [60].

Piezoelectric biosensors, commonly known as quartz crystal microbalance (QCM) biosensors, detect mass changes on a biosensor surface by measuring variations in the frequency of a quartz crystal or other piezoelectric materials. These biosensors are highly sensitive to mass changes and offer real-time monitoring capabilities. During viral infections such as Influenza A, piezoelectric biosensors have been used to detect cytokines like Interleukin-6 (IL-6). These biosensors have achieved a LOD of 0.5 pg/mL, making them effective for monitoring cytokine levels during the acute phase of infection [61–63].

These biosensors are essential tools for detecting and monitoring biomarkers associated with viral infections, providing high sensitivity and specificity, and enabling real-time assessment of disease progression.

### Nanobiosensors

Biosensors, which include a receptor sensor and a detector, are designed to convert biological signals into digital outputs. Their efficiency is significantly enhanced by nanomaterials such as nanofilms [64], gold nanoparticles [65, 66], and quantum dots [67], which amplify the signals. Nanomaterials possess exceptional optical, electrical, magnetic, and mechanical properties, as well as high surface area-to-volume ratios. These attributes make them ideal for minimal invasive techniques and enable the rapid, sensitive detection of viruses. Moreover, nanobiosensors facilitate real-time monitoring, a crucial capability for improving patient care by providing timely and accurate diagnostics, even at low viral levels. Their enhanced sensitivity allows for the detection of even minimal viral quantities, ensuring timely and accurate diagnostics.

**Fig. 2:**
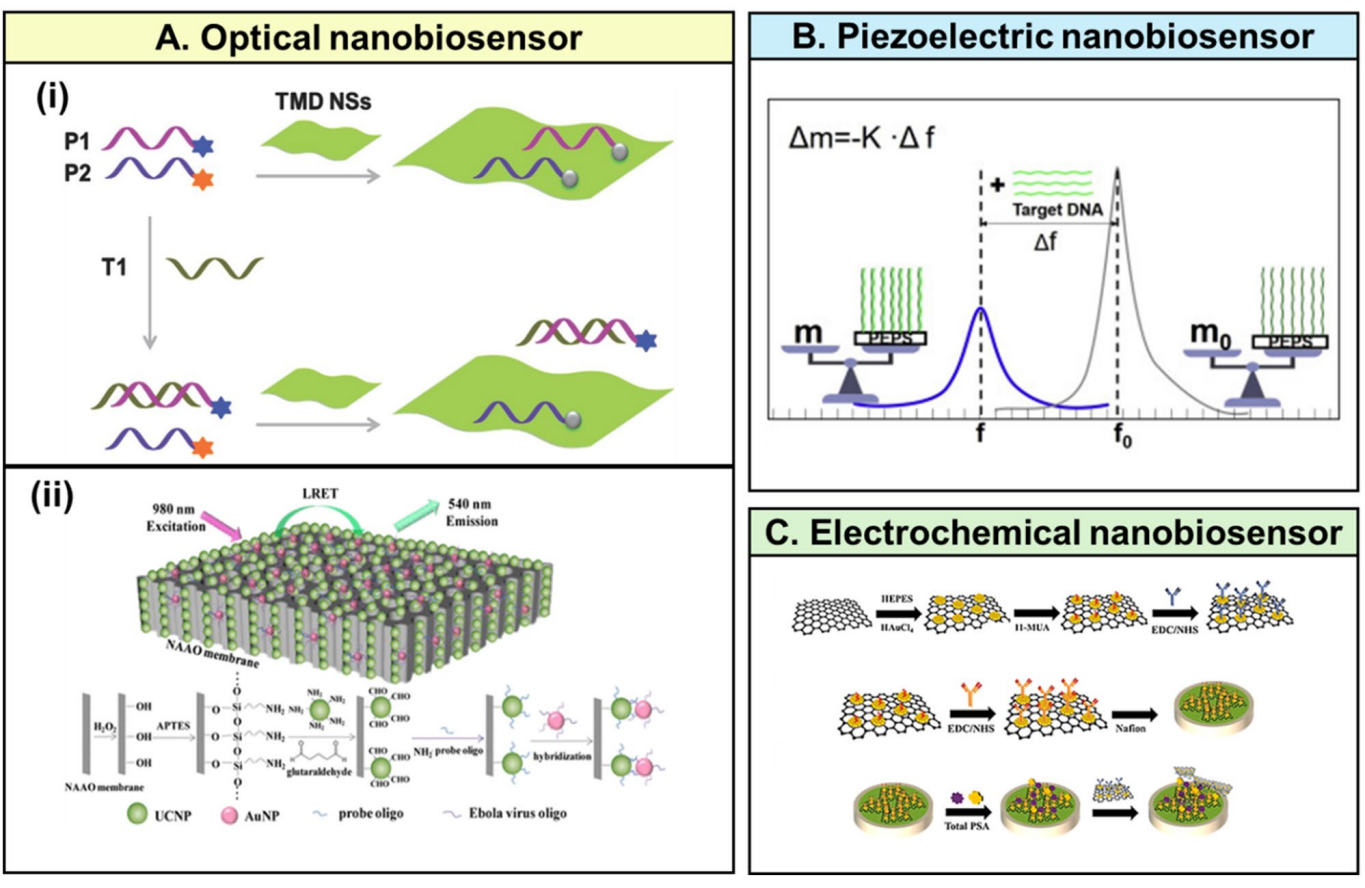
Applications of biosensors for viral detection. (**A**) Optical nanobiosonsers. (i) Single-layer transition metal dichalcogenide nanosheets-based multiplexed fluorescent DNA detection [75]. Reprinted with permission granted by John Wiley and Sons via Copyright Clearance Center, Inc. (ii) Oligo detection based on luminescence resonance energy transfer (LRET) biosensor with energy transfer from upconversion nanoparticles (UCNPs) to gold nanoparticles (AuNPs) on membrane [77]. Reprinted with permission from Copyright 2016 American Chemical Society. (**B**) Piezoelectric plate nanobiosensor [80]. Reprinted with permission granted by Elsevier via Copyright Clearance Center, Inc. (**C**) Novel electrochemical sensor for prostate-specific antigen marker detection [81]. Reprinted with permission granted by John Wiley and Sons via Copyright Clearance Center, Inc

Viral infections often induce metabolic changes in both infected and responding host cells, making these alterations potential indicators for immediate monitoring of viral activity [68]. Commonly used nanobiosensors for monitoring virus infection include optical [69, 70], piezoelectric [71, 72], and electrochemical [73, 74] nanobiosensors (Fig. 2).

Optical nanobiosensors have emerged as a leading technology in nano-biosensing due to their noninvasiveness, high sensitivity, direct detection capabilities, and ease of integration with other technologies. Leveraging their inherent properties, optical nanobiosensors enable early diagnosis and enhance clinical outcomes. The fundamental type of optical nanobiosensor is an optical transducer, which detects analytes or pathogens by measuring changes in fluorescence, absorption, or reflectance of the sensing material. For virus detection, optical nanobiosensors are often combined with organic fluorescent molecules [75], quantum dots (QDs) [76], upconversion nanoparticles (UCNPs) [77], gold nanoparticles (AuNPs) [78], and magnetic particles (MPs)-chemiluminescent labels [79].

Piezoelectric nanobiosensors function by measuring changes in resonance frequency. Quartz crystal microbalances (QCMs), which have piezoelectric properties, detect these frequency changes or deflections in the sensing material. Piezoelectric materials experience mechanical oscillations when subjected to an alternating current (AC) voltage, creating an oscillating electric field. The frequency (f) of the AC voltage decreases as the mass (m) increases due to intermolecular interactions [80]. Mass-responsive piezoelectric nanobiosensors are widely used for virus detection. In these nanobiosensors, an antibody is immobilized on the upper electrode surface of the piezoelectric material. The upper and lower electrodes induce resonance in the piezoelectric material. When the target antigen binds to the probe antibody, the resonance frequency changes, allowing detection.

Electrochemical nanobiosensors detect changes in electrical signals and can be classified into amperometric, potentiometric, or conductivity sensors [81]. Amperometric sensors apply a voltage between a reference and working electrode to initiate electrochemical oxidation or reduction, quantifying analyte concentration by measuring the resulting current. Potentiometric sensors, through specific sensor-analyte interactions, establish a local Nernstian equilibrium at the sensor interface, providing analyte concentration information without current flow. Conductometric sensors, also known as impedimetric sensors, detect and quantify analyte-specific recognition events by measuring changes in surface impedance on the electrode.

In addition to monitoring viral load, it is crucial to examine the role of nanobiosensors in assessing cytokine impact during viral infections. Nanobiosensors offer valuable insights into both viral load and cytokine levels, which are key modulators of the immune response during infections. Understanding this interplay is essential for developing comprehensive diagnostic tools and therapeutic strategies.

## OoC models for viral infection

### OoC models

OoC models have transformed biomedical research by offering dynamic platforms that simulate human organ systems, providing valuable insights into viral infections and responses to antiviral drugs. The COVID-19 pandemic has notably accelerated their application in virological research, making them essential tools for studying viral pathogenesis and testing therapeutic interventions (Table 1) [82, 83]. To prepare for future pandemics, expanding OoC models to include a wider variety of organs and a broader range of virus strains is crucial for improving our understanding of viral infections and treatment responses.

#### Lung-on-chip

**Fig. 3:**
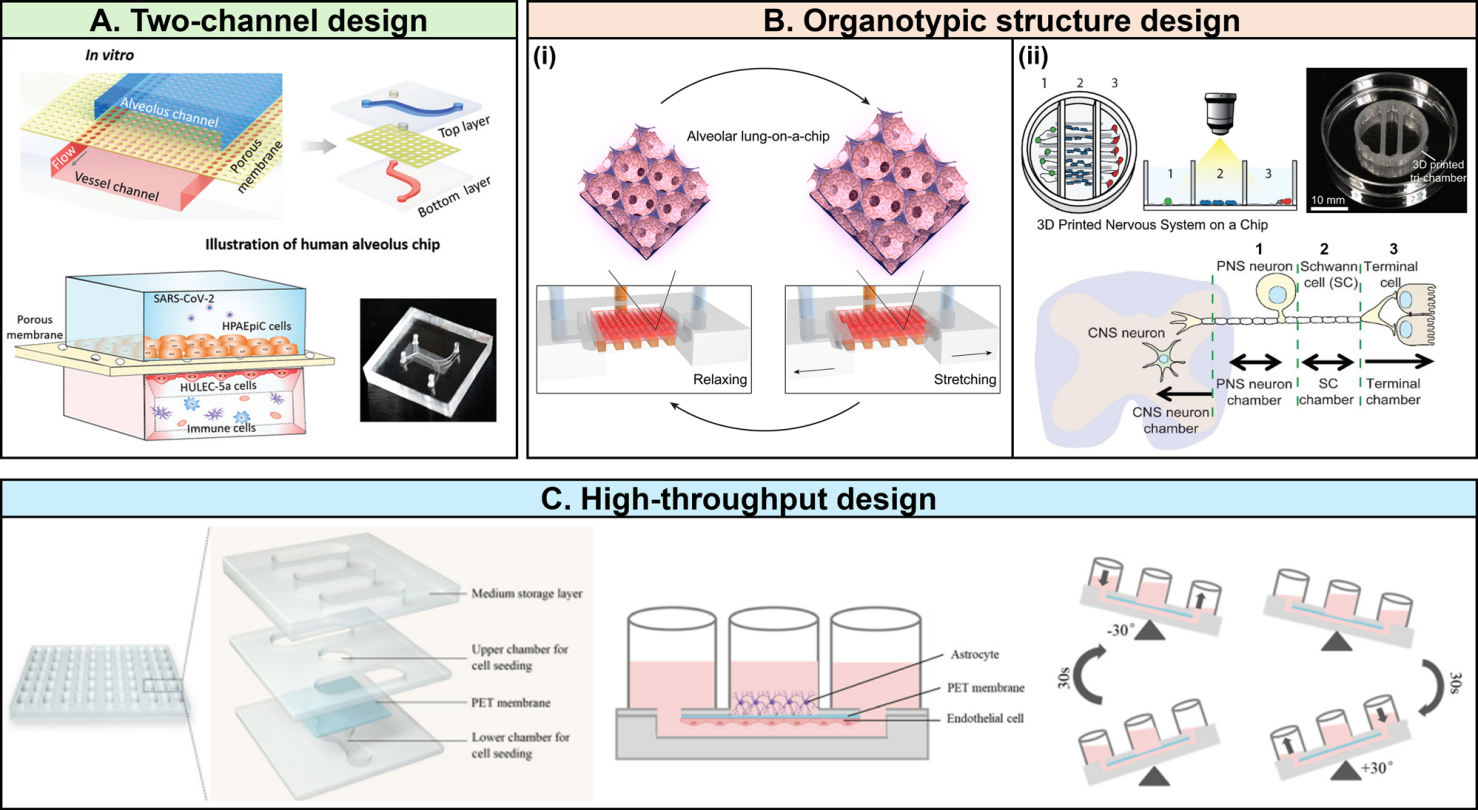
Various designs of Organ-on-Chip (OoC) systems for replicating viral infections in human organs. (**A**) Lung-on-Chip model with two parallel channels separated by a porous cell culture membrane [85]. Reprinted under the terms of the Creative Commons CC-BY license. (**B**) OoCs incorporating organotypic structures. (i) Lung-on-Chip featuring alveolar sac-like structure made from gelatin methacryloyl (GelMA) hydrogel [94]. Reprinted under the terms of the Creative Commons Attribution-NonCommercial-NoDerivatives License 4.0 (CC BY-NC-ND) license. (ii) Nervous system-on-Chip with three chambers culturing different type of cells [101]. Reprinted with permission granted by RSC Publishing via Copyright Clearance Center, Inc. (**C**) High-throughput Blood-brain barrier (BBB)-on-Chip, operating multiple organ-on-chip units in a single array [103]. Reprinted with permission granted by RSC Publishing via Copyright Clearance Center, Inc

Lung-on-Chip models have been extensively used to study respiratory infections, especially SARS-CoV-2. These models often employ microfluidic devices with two parallel channels separated by porous membranes to mimic the alveolar-capillary interface [84–92]. An advanced design includes lateral vacuum chambers that replicate the mechanical stress of breathing, providing a more physiologically relevant environment for studying lung infections [93]. Additionally, 3D structures, such as porous gelatin methacryloyl (GelMA) scaffolds, are used to replicate alveolar sacs, enhancing the model’s ability to mimic in vivo lung architecture and function during viral infection studies (Fig. 3) [94].

#### Liver-on-chip

Liver-on-Chip models are invaluable for studying hepatotropic viruses, such as the Hepatitis B Virus (HBV). These models are designed to replicate the liver’s complex microarchitecture, often using microfluidic systems that simulate liver tissue perfusion and metabolic functions [95–97]. Advanced platforms, such as those incorporating a capillary bed structure, allow for more accurate modeling of viral infection dynamics and drug metabolism. High-throughput liver models have been developed to improve usability and scalability, specially for screening antiviral drugs targeting liver infections [97].

#### Kidney-on-chip

Kidney-on-Chip models focus on mimicking the renal filtration barrier, which is crucial for studying viral infections that impact kidney function [12]. These models commonly use two-channel microfluidic devices with a porous membrane that simulates the glomerular filtration barrier. This configuration allows researchers to investigate how viruses affect kidney health and renal filtration, as well as to assess potential nephrotoxic effects of antiviral drugs.

#### Gut-on-chip

Gut-on-Chip models are designed to replicate the intestinal barrier, often using two-channel microfluidic devices with porous membranes designed to mimic the epithelial-endothelial interface [98–100]. Some designs also incorporate mechanical features that simulate peristaltic movement, enhancing their physiological relevance. These models are used to study enteric viruses, such as coxsackievirus and coronavirus, allowing researchers to explore viral replication dynamics and the gut’s immune response.

#### Brain-on-chip

Brain-on-Chip models are particularly useful for studying viral infections that affect the central nervous system, such as neurotropic viruses. These models typically simulate the blood-brain barrier (BBB) through complex microfluidic systems, including hollow cylindrical channels lined with brain endothelial cells [101–104]. Certain advanced designs include multiple chambers that house different brain cell types, allowing researchers to examine interactions between cells and the effects of viral infections on brain function [101]. High-throughput BBB-on-chip models have been developed to facilitate studies on viral penetration and drug delivery across the BBB [103].

**Table 1:** Viral infection models in human organ-on-chips

Organ	Design	Cell type	Viral strain	Finding	Ref.
Lung	Two channels separated by a porous membrane	Primary human alveolar epithelial cells (pHAECs), primary human lung endothelial cells (pHLECs)	H1N1, H3N2, H5N1	- Influenza infection in human lung. - Inhibition TRPV4 and RAGE in influenza infection.	[84]
	Two channels separated by a porous membrane	human alveolar epithelial cell (hAECs), HULEC-5a	SARS-CoV-2	- Infection in human lung. - Effects of Remdesivir on infection.	[85]
	Three channels (middle: collagen, side: cells)	pHAECs, human umbilical vein endothelial cells (HUVEC)	SARS-CoV-2	- Recapitulating SARS-CoV-2 infection in human lung - Effects of spike protein-specific antibody on preventing infection.	[86]
	Two channels separated by a porous membrane	pHAECs, pHLECs	SARS-CoV-2	- Damages in lung endothelium by infection. - Effects of Tocilizumab and anti-IL6R antibody, on endothelial cell damages.	[87]
	Two channels separated by a porous membrane	pHAECs, pHLECs	SARS-CoV-2	- cGAS–STING Pathway drives type I IFN immunopathology in COVID-19.	[88]
	Incorporated with an alveolar sac-like porous scaffold	pHAECs	SARS-CoV-2	- Infection of human lung.	[94]
	Two channels separated by a porous membrane	pHAECs, pHPECs	H1N1	- Emergence of drug-resistant viral strains by chip-to-chip transmission under presence of drugs.	[89]
	Two channels separated by a porous membrane	Primary human lung bronchial-airway epithelial basal stem cells, pHPECs	H1N1, H3N2, H5N1, SARS-CoV-2	- Viral infections and their treatments.	[90]
	Two channels separated by a porous membrane	pHAECs, pHLECs	Viral mimic (poly(I: C))	- Exacerbation of chronic obstructive pulmonary disease by viral infection	[91]
	Two channels separated by a porous membrane	pHAECs, HUVECs, neutrophil	Human rhinoviral 16	- Asthma exacerbation and transepithelial migration.	[92]
Liver	Single channel or two channels separated by a porous membrane	Primary rat hepatocytes, primary rat adrenal medullary endothelial cells, bovine aortic endothelial cells (bAECs)	Hepatitis B	- Viral replication.	[95]
	Single channel	Primary human hepatocytes (pHHs), bAECs	Hepatitis B	- Viral replication.	[96]
	incorporating scaffold that contains matrix mimicking liver capillary bed	Human hepatocyte cell lines, pHHs, primary Kupffer cells	Hepatitis B	- Infection.	[97]
Gut	Two channels separated by a porous membrane	Caco-2 (human gut epithelial cell line)	Coxsackieviral B1	- Infection and corresponding responses are polarized in gut epithelial cells.	[98]
	Two channels separated by a porous membrane	Caco-2, HUVECs	SARS-CoV-2	- Infection in human gut.	[99]
	Two channels separated by a porous membrane	Organoids-derived gut epithelial cells, primary human large intestine microvascular endothelial cells	HCoV-NL63	- Infection and its treatment in human gut.	[100]
Kidney	Single channel	Madin Darby Canine Kidney cells	Pseudorabies viral	- Pseudorabies viral-induced abnormalities	[12]
Brain	Three chambers connected with each other via microchannels	Primary rat neurons, S16 (rat Schwann cell line), PK-15 (porcine kidney cell line)	Pseudorabies viral	- Connectivity among the individual cellular components through axonal viral transport.	[101]
	Hollow cylindrical channel in hydrogel	human brain endothelial cell line (hCMEC/D3)	SARS-CoV-2 spike protein S1	- Effects of SARS-CoV-2 spike protein on BBB integrity.	[102]
	Chamber and single channel separated by a porous membrane	hCMEC/D3, primary human astrocytes	Adeno-associated viral	- Adeno-associated viral translocation mediated by LY6E protein crossing human BBB.	[103]
	Two channels separated by a porous membrane	Primary human brain endothelial cells, primary human pericytes, SVGp12 (human astrocyte cell line)	Venezuelan Equine Encephalitis virals	- Long-term infection in BBB. - Effectiveness of Omaveloxolone.	[104]

### Evaluating antiviral drug efficacy and infection phenotypes using OoCs

OoC models are essential for evaluating antiviral drug efficacy by examining various infection phenotypes, including cytokine release, marker protein expression, viral binding, and replication [84, 85, 87, 88, 90, 92]. Some studies have focused on noninvasive methods for assessing these phenotypes, such as phase contrast microscopy for cell morphology and trans-epithelial/endothelial electrical resistance (TEER) measurement for barrier integrity [98, 100]. Unique applications of OoCs include using Lung-on-Chip models to investigate the evolution of influenza A virus and the emergence of drug-resistant strains [89]. Additionally, OoCs have been employed to explore interactions between different cell types during infection, as demonstrated in models simulating viral-induced asthma exacerbations and brain infection dynamics [92, 101].

These models not only elucidate the direct effects of antiviral drugs on viruses but also identify potential side effects on host cells, offering a comprehensive assessment of drug efficacy and safety. As OoC technology continues to evolve, it holds the potential to significantly enhance our understanding of viral infections and the development of effective therapeutic strategies.

## Integration of nanobiosensors into OoC models

### Designing factors of integration of nanobiosensors into OoC models

The integration of nanobiosensors into OoC models for real-time monitoring represents an emerging yet underexplored frontier with considerable potential to advance virological and biomedical research. Real-time monitoring is critical for investigating dynamic processes, including cytokine storms, fluctuations in barrier integrity, and the progression of viral infections. Traditional models require sample collection for biosensor analysis, often leading to interruptions and potential data loss. By embedding nanobiosensors directly within OoCs, researchers can achieve continuous, real-time tracking of key biomarkers without system disruption, enabling a more precise and holistic understanding of these biological phenomena [105]. The compact design of nanobiosensors allows for seamless integration with OoCs [13–15], while their high sensitivity facilitates the detection of cytokines at low concentrations, significantly enhancing LOD beyond that of conventional biosensors [62, 63].

**Fig. 4:**
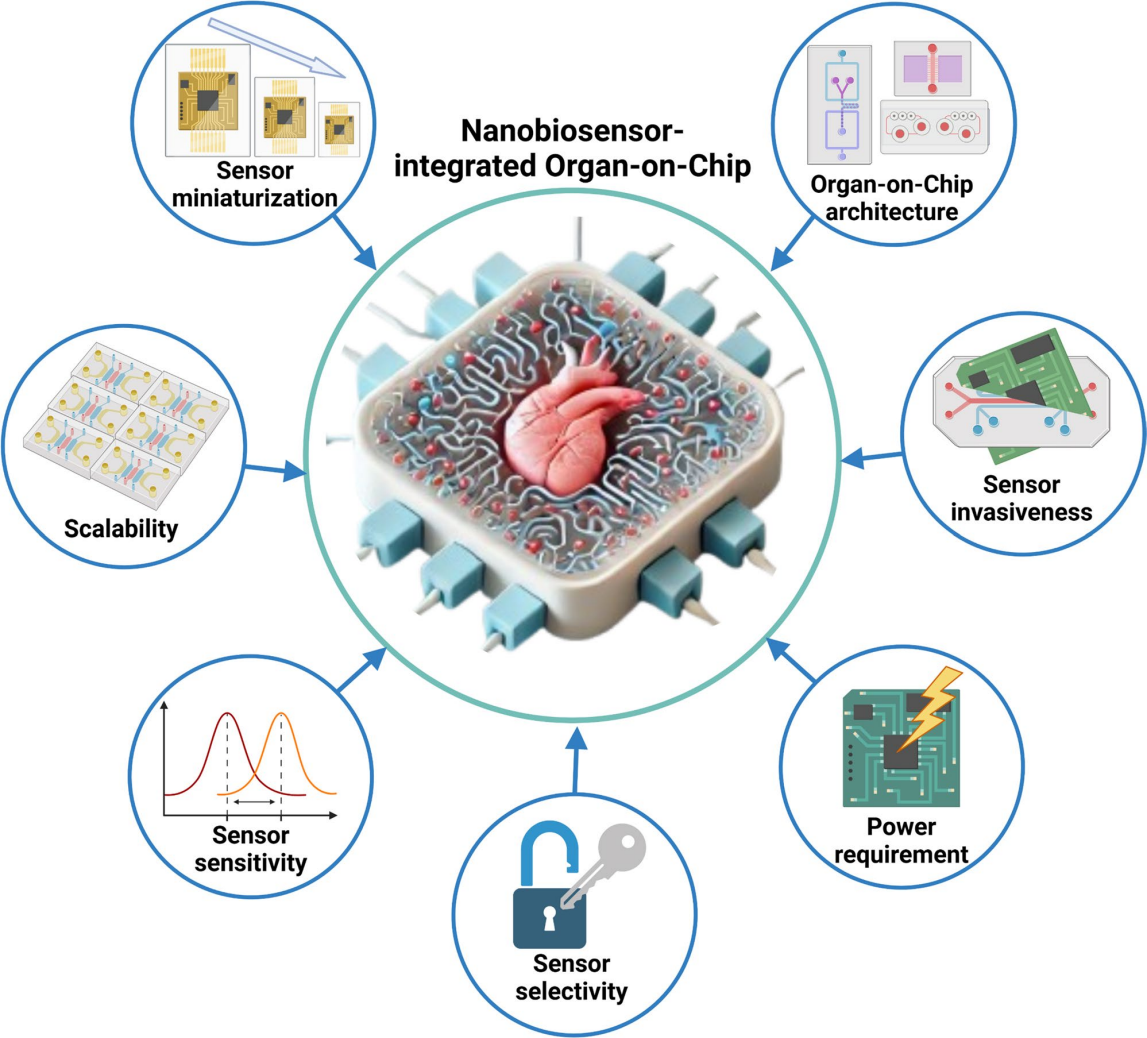
Design factors for establishing nanobiosensor-integrated organ-on-chip. Sensor miniaturization ensures that nanobiosensors can fit within the microfluidic channels and compartments of OoCs without disrupting the biological environment. Scalability supports efficient, cost-effective production of nanobiosensors for a range of OoC platforms. Sensor sensitivity enables the detection of low concentrations of biomarkers, such as cytokines, providing accurate real-time data on immune responses. Sensor selectivity allows nanobiosensors to target specific biomarkers, ensuring specificity and minimizing cross-reactivity. Power requirement addresses the need for low-power designs to reduce heat generation and energy usage. Sensor invasiveness emphasizes the importance of noninvasive sensing to prevent disruption to biological processes on the chip. Organ-on-chip architecture ensures that nanobiosensors are strategically integrated within the microfluidic structure to maintain optimal fluid flow and effective sample contact, supporting continuous, multiplexed monitoring for comprehensive virological and biomedical research. (Created with Biorender.com)

When incorporating nanobiosensors into OoC models, several critical design considerations must be addressed to ensure effective functionality and seamless operation (Fig. 4). Given the micro-scale dimensions of OoC systems, nanobiosensors must be sufficiently miniaturized to fit within the microfluidic channels and compartments of the chip. Additionally, these nanobiosensors should not interfere with the normal physiological functions of the tissues or cells within the OoC. Compatibility with materials commonly used in OoCs, such as polydimethylsiloxane (PDMS) and glass, is also essential to avoid any adverse interactions that could affect nanobiosensors performance or alter the biological environment [106].

Sensitivity and selectivity are crucial aspects of nanobiosensor design. Nanobiosensors must exhibit high sensitivity to detect low concentrations of specific biomarkers, such as cytokines or viral particles, while avoiding cross-reactivity with other substances in the OoC environment. This necessitates careful selection and engineering of sensing elements, such as antibodies or aptamers, to ensure they bind exclusively to the target molecules [107]. Additionally, nanobiosensors must support real-time, continuous monitoring to provide meaningful insights into the dynamic processes within the OoC. They should be capable of operating over extended periods without degradation to ensure consistent and reliable data on biomarker levels. Integration with data acquisition systems that enable continuous data collection and analysis is also essential [105].

Noninvasiveness is a crucial factor to consider. To minimize disruption to the biological processes within the OoC, it is important to prioritize noninvasive or minimally invasive sensing technologies. These technologies facilitate the monitoring of biomarkers without causing physical damage or altering the behavior of the cells and tissues on the chip [108]. Additionally, in many cases, it is necessary to monitor multiple biomarkers simultaneously to achieve a comprehensive understanding of the biological processes within the OoC. Therefore, nanobiosensors with multiplexing capabilities are essential, as they enable the detection of several different biomarkers within the same chip and provide a more complete view of disease progression, drug effects, or viral infection dynamics.

Integration of nanobiosensors into the microfluidic architecture of the OoC is a critical design consideration. This requires careful placement of nanobiosensors within the chip to ensure optimal fluid flow and effective contact with the biological sample. The design must support efficient delivery and removal of fluids and accommodate nanobiosensors arrays without disrupting the microfluidic environment [109]. Additionally, data integration and analysis should be integral to the design. The system needs to incorporate mechanisms for data collection, storage, and analysis, potentially by integrating nanobiosensors with external data processing units or embedding data processing capabilities within the OoC. Advanced data analysis techniques, such as machine learning algorithms, can be used to interpret the complex datasets generated by the nanobiosensors [110, 111].

Power requirements and signal transduction mechanisms are also critical design considerations. Low-power designs are preferred to reduce heat generation and energy consumption within the OoC. The signal transduction mechanism should be optimized to minimize noise and ensure precise data transmission from the nanobiosensors to external devices for analysis [112]. Additionally, the materials used in constructing the nanobiosensors must be biocompatible to avoid adverse reactions with the biological components of the OoC. The nanobiosensors should also be durable enough to maintain reliable performance throughout long-term experiments, without degradation.

Scalability and manufacturability are key considerations in the design of nanobiosensors for OoC models. It is essential to develop nanobiosensors that can be produced at scale and integrated into OoCs both efficiently and cost-effectively. The design should also accommodate adaptation to different OoC platforms and applications, ensuring broad applicability across various research fields. For instance, electrochemical nanobiosensors could be integrated into Lung-on-Chip models to monitor cytokines like IL-6 or TNF-α during a viral infection. These nanobiosensors can provide immediate feedback on inflammatory responses, allowing researchers to observe how these levels fluctuate in response to infection or drug treatment. Similarly, optical nanobiosensors, such as those utilizing SPR, can detect real-time changes in the concentration of specific proteins or antibodies in Liver-on-Chip or Gut-on-Chip models.

### Recent advances of nanobiosensors-integrated OoC models

**Fig. 5:**
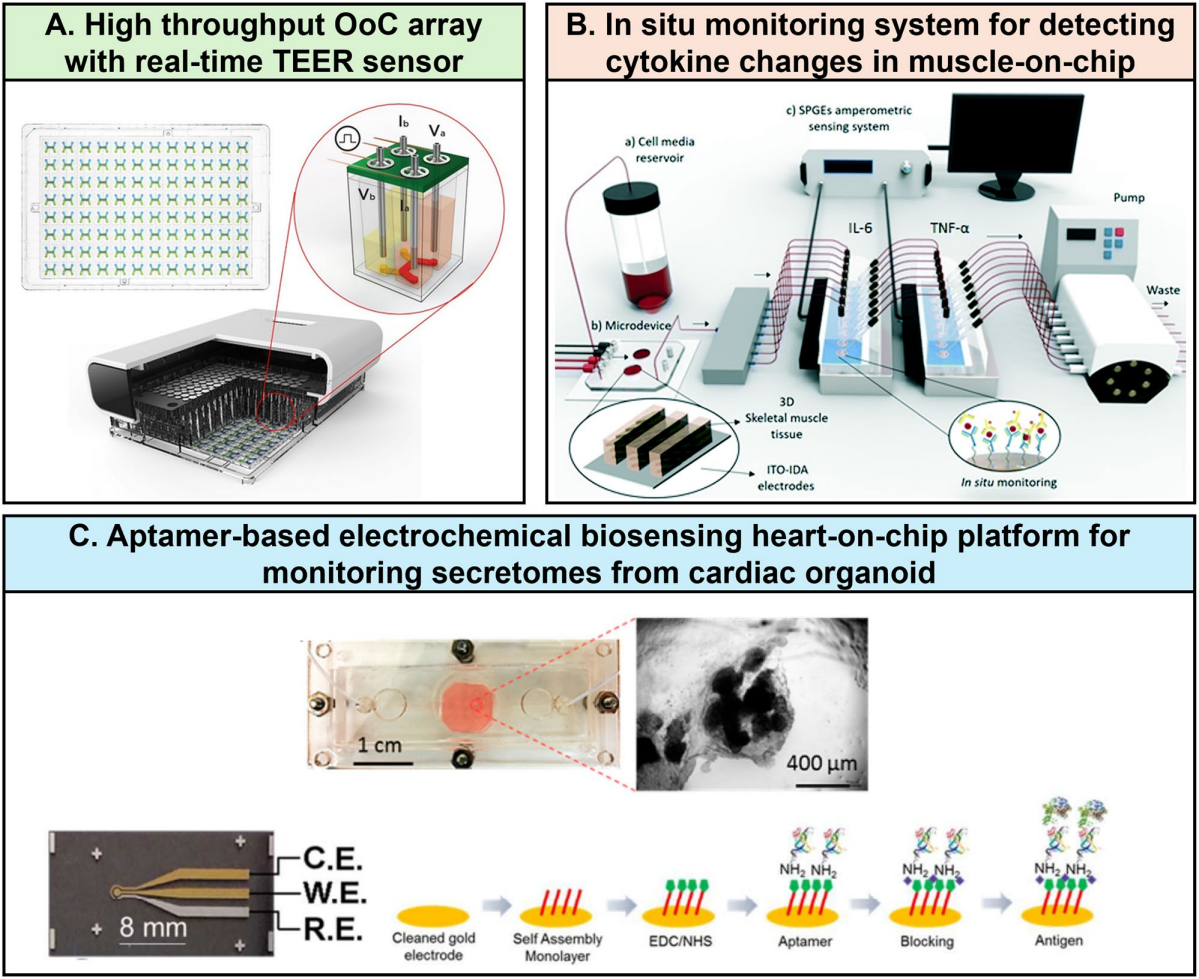
Integration of biosensors in organ-on-chip systems for in situ and real-time monitoring of changes. (**A**) OoC arrays equipped with high-throughput trans-epithelial/endothelial electrical resistance (TEER) sensor for real-time non-invasive monitoring of nephrotoxicity [114]. Reprinted under the terms of the Creative Commons CC-BY license. (**B**) In situ monitoring system for detecting IL-6 and TNF-α changes in a muscle-on-chip [117]. Reprinted with permission granted by RSC Publishing via Copyright Clearance Center, Inc. (**C**) Heart-on-chip equipped with aptamer-based biosensor to monitor cell-secreted trace cardiac biomarkers [120]. Reprinted with permission from Copyright 2016 American Chemical Society

Integrating nanobiosensors into OoCs represents a particularly promising area of research. Nanobiosensors can greatly enhance these models by enabling time-resolved, continuous measurements and providing faster readouts, which results in more comprehensive data from organ and disease models (Fig. 5; Table 2). For example, Asif et al. integrated optical pH nanobiosensors and TEER electrodes into a glass-based kidney-on-chip to monitor real-time changes under disease conditions and drug treatment. They demonstrated that decreases in pH and TEER values over time served as noninvasive indicators of renal damage [113]. Similarly, Shaughnessey et al. employed high-throughput TEER measurement in an OoC array to detect drug-induced nephrotoxicity, showing that changes in TEER could be observed before cell death, offering a rapid, early, and label-free approach to toxicity assessment [114]. The integration of electrochemical immunosensors into OoC platforms enables the use of various techniques, such as voltammetry, amperometry, and impedance measurements, to enhance signal and nanobiosensors sensitivity [115]. Real-time detection of molecules secreted by cells, such as cytokines, is crucial for monitoring changes in cellular and tissue metabolic activity, particularly during immune responses to viral infections. Cytokines regulate various cellular functions during immune responses, including cell proliferation, migration, or activation, and play a key role in viral infection and intercellular signaling [116]. The most commonly used in situ techniques for detecting cytokines and small proteins on microarrays are immunoassay-based methods, such as antigen-antibody assays [117]. Regarding the integration of antibody-based nanobiosensors into OoC platforms, various functionalization strategies have been developed to ensure efficient and stable immobilization of antibodies on nanobiosensors electrodes, thus enhancing detection sensitivity [118, 119]. For example, Ortega et al. developed a PDMS-based muscle microarray platform for monitoring inflammatory cytokines IL-6 and TNF-α produced by mouse skeletal myoblasts [117]. Aptamers are also gaining popularity for detecting proteins and cytokines due to their high sensitivity, selectivity, and thermal stability. Shin et al. developed an aptamer-based electrochemical biosensing platform connected to a bioreactor culture chamber for monitoring damage in cardiac-like organs [120]. Additionally, Liu et al. quantified IFN-γ and TNF-α production by activated T cells in a microfluidic device by labeling aptamers with anthraquinone and methylene blue redox reporters on gold electrodes [121].

**Table 2:** Nanobiosensors-integrated human organ-on-chips

Type	Application	Biomarkers	Organ	Context of detection	Ref.
Optical	Tissue damage and pH monitoring	pH	Kidney	- Renal damage under disease and drug treatment conditions.	[113]
Electrical	Muscle inflammation	IL-6, TNF-α	Muscle	- Real-time monitoring of inflammatory cytokines produced by muscle cells under inflammatory conditions.	[117]
Electrical	Cardiovascular disease	Cardiac Electrophysiology	Heart	- Electrophysiological responses to hypoxia on heart.	[118]
Electrochemical	Cardiac tissue damage	Cardiac damage markers	Cardiac	- Monitoring cardiac damage markers in real time.	[120]
Electrochemical	Immune response and cytokine quantification	IFN-γ, TNF-α	T cells	- Quantifies cytokine production by T cells.	[121]

### Future directions of nanobiosensor-integrated OoCs

Looking forward, the integration of nanobiosensors in multi-organ-on-chip (MoC) systems holds tremendous potential for studying systemic viral responses across interconnected organ models. MoCs enable a more comprehensive understanding of multi-organ interactions in diseases where viral infections affect multiple systems, such as the respiratory, hepatic, and neural tissues. For example, a gut-liver MoC equipped with nanobiosensors could simultaneously track gut permeability and liver responses, providing real-time insights into the cross-organ infection dynamics. However, designing nanobiosensors that are compatible with diverse cell types and environments across MoCs remains a challenge, often requiring modular configurations where each organ can be monitored independently before being connected into an integrated system.

Artificial intelligence (AI) and machine learning (ML) are essential for managing and interpreting the large-scale data generated by nanobiosensor-integrated OoCs, especially as multi-organ setups introduce additional complexity. ML algorithms excel at handling high-dimensional datasets, .identifying patterns, predictive markers, and anomalies critical for studying viral pathogenesis and therapeutic effects. Additionally, ML-driven image recognition within these systems can track cellular changes in real time, while label-free virtual staining techniques allow uninterrupted visualization of cellular structures, preserving the integrity of biological data [122].

Beyond data analysis, AI plays a crucial role in optimizing operational conditions within nanobiosensor-integrated OoCs. Real-time AI control of factors like media flow and environmental stability ensures the maintenance of optimal conditions, reducing manual adjustments and enhancing data reliability [110, 111]. Advances in bioprinting and AI-supported design are also facilitating the production of complex, fully automated OoC systems with embedded nanobiosensors, allowing for efficient, safe, and reproducible experimental workflows, especially in studies involving infectious pathogens.

**Fig. 6:**
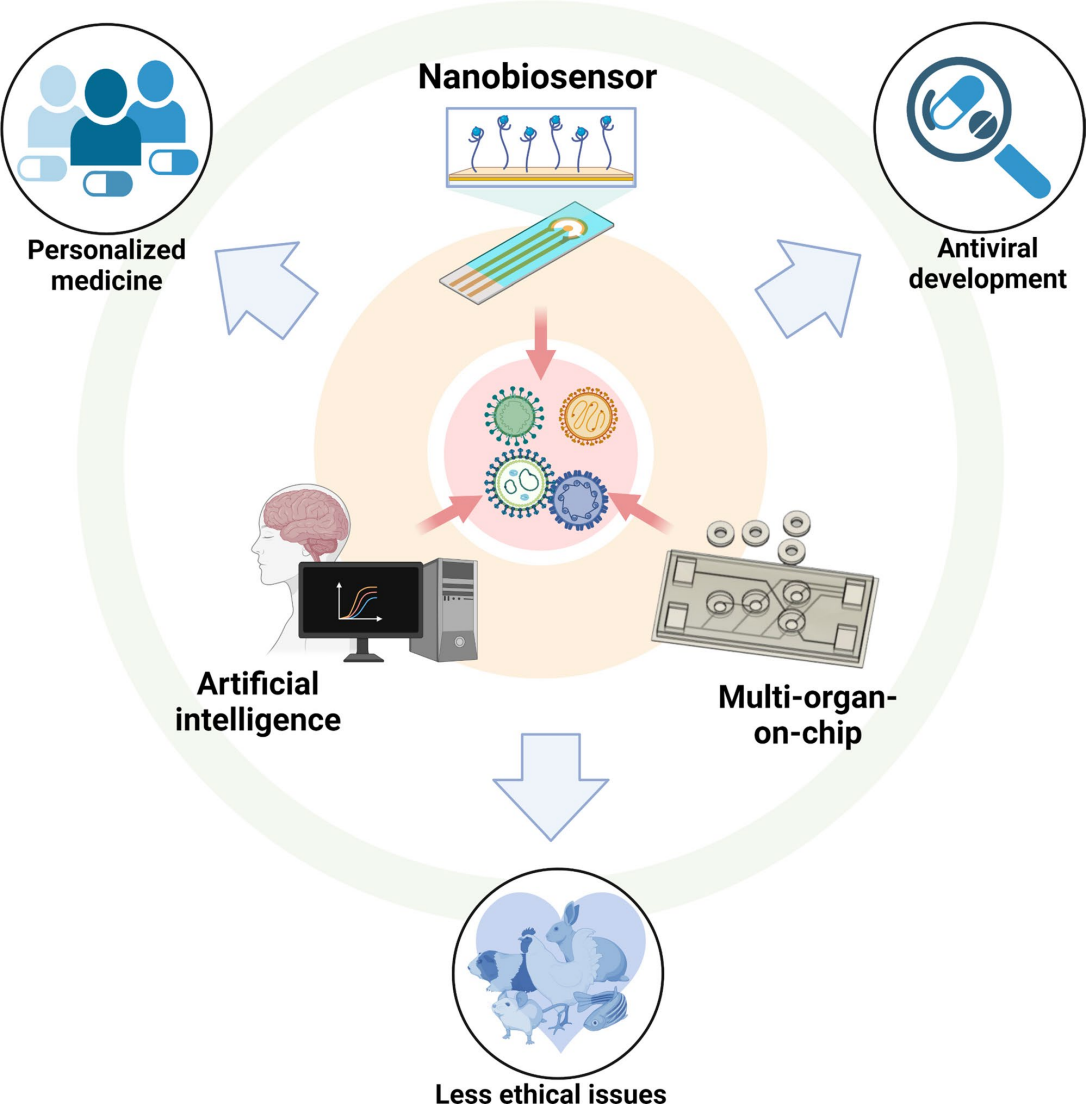
Potential integration of advanced technologies in organ-on-chip systems and its potential outcomes in virological research. Nanobiosensor enables real-time monitoring of biomarkers, essential for tracking viral infection dynamics and immune responses. Multi-organ-on-chip systems mimic interactions across different organs, enhancing the physiological relevance of disease models. Artificial intelligence aids in data analysis and interpretation of complex datasets generated by organ-on-chips. These integrations collectively support personalized medicine and antiviral development and reduce ethical concerns by minimizing reliance on animal testing. (Created with Biorender.com)

The future of nanobiosensor-integrated OoCs, combined with the capabilities of MoCs and AI, promises a transformative approach in virological research and personalized medicine. These innovations not only enable precise, real-time monitoring of multi-organ interactions but also support a new level of data accuracy and more ethical research practices by reducing reliance on animal models, ultimately contributing to more effective and patient-centered healthcare solutions (Fig. 6).

## Conclusion

Integrating nanobiosensors into OoC systems offers substantial potential for advancing our understanding of viral infections and enhancing therapeutic interventions. Nanobiosensors overcome key limitations of traditional diagnostic methods by enabling real-time, in situ monitoring of biomarkers such as cytokines and other disease indicators. Their miniaturized design, increased sensitivity, and compatibility with microfluidic environments make nanobiosensors ideal for continuous, noninvasive monitoring. This capability provides researchers with critical insights into the dynamic processes of viral infections and immune responses, aiding the development of more effective treatments.

However, despite advancements, significant challenges remain in fully integrating nanobiosensors into OoCs, which limits these system’s ability to replicate the complex, dynamic nature of human biology. Future research should prioritize addressing these challenges, including nanobiosensors durability, multiplexing capabilities, and data analysis. The continued development of nanobiosensors-integrated OoCs will revolutionize virological research by enabling real-time biomarker monitoring, improving drug dosing precision, and facilitating early detection of adverse effects.

In conclusion, the synergy between OoCs and nanobiosensors is set to revolutionize virological research and therapeutic development. Ongoing advancements in materials science, multi-organ chip designs, and data processing will drive the scalability and functionality of these integrated systems. As these technologies progress, they hold the potential to unlock new possibilities for personalized medicine, real-time diagnostics, and more effective treatments for viral diseases.

## Data Availability

Not applicable.

## Abbreviations

OoC: Organ-on-chip
2D: Two dimensional
IL-6: Interleukin-6
CRP: C-reactive protein
TNF-α: Tumor Necrosis Factor-alpha
IL-1β: Interleukin-1 beta
IFN-γ: Interferon-gamma
ALT: Alanine aminotransferase
AST: Aspartate aminotransferase
LDH: Lactate dehydrogenase
WBC: White blood cell
LOD: Limit of detection
HBV: Hepatitis B virus
SPR: Surface plasmon resonance
QCM: Quartz crystal microbalance
QD: Quantum dot
UCNP: Upconversion nanoparticle
AuNP: Gold nanoparticle
MP: Magnetic particle
AC: Alternating current
GelMA: Gelatin methacryloyl
BBB: Blood-brain barrier
TEER: Trans-epithelial/endothelial electrical resistance
PDMS: Polydimethylsiloxane
AI: Artificial intelligence
MoC: Multi-organ-on-chip
ML: Machin learning

## References

[1] A. Schweitzer, J. Horn, R.T. Mikolajczyk, G. Krause, J.J. Ott, Estimations of worldwide prevalence of chronic hepatitis B virus infection: a systematic review of data published between 1965 and 2013. Lancet. **386**(10003), 1546–1555 (2015). 10.1016/S0140-6736(15)61412-X 26231459 10.1016/S0140-6736(15)61412-X

[2] B. Ackerson, H.F. Tseng, L.S. Sy, Z. Solano, J. Slezak, Y. Luo, C.A. Fischetti, V. Shinde, Severe morbidity and mortality associated with respiratory syncytial virus versus influenza infection in hospitalized older adults. Clin. Infect. Dis. **69**(2), 197–203 (2019). 10.1093/cid/ciy991 30452608 10.1093/cid/ciy991 PMC6603263

[3] I. Adetifa, J.-J. Muyembe, D.G. Bausch, D.L. Heymann, Mpox neglect and the smallpox niche: a problem for Africa, a problem for the world. Lancet. **401**(10390), 1822–1824 (2023). 10.1016/S0140-6736(23)00588-3 37146622 10.1016/S0140-6736(23)00588-3 PMC10154003

[4] N. Ashammakhi, R. Nasiri, N.R. Barros, P. Tebon, J. Thakor, M. Goudie, A. Shamloo, M.G. Martin, A. Khademhosseini, Gut-on-a-chip: current progress and future opportunities. Biomaterials. **255**, 120196 (2020). 10.1016/j.biomaterials.2020.120196 32623181 10.1016/j.biomaterials.2020.120196 PMC7396314

[5] H. Page, P. Flood, E.G. Reynaud, Three-dimensional tissue cultures: current trends and beyond. Cell. Tissue Res. **352**(1), 123–131 (2013). 10.1007/s00441-012-1441-5 22729488 10.1007/s00441-012-1441-5

[6] T. Hartung, Toxicology for the twenty-first century. Nature. **460**(7252), 208–212 (2009). 10.1038/460208a 19587762 10.1038/460208a

[7] S. Cho, S. Lee, S.I. Ahn, Design and engineering of organ-on-a-chip. Biomed. Eng. Lett. **13**(2), 97–109 (2023). 10.1007/s13534-022-00258-4 10.1007/s13534-022-00258-4 PMC9806813 36620430

[8] Q. Wu, J. Liu, X. Wang, L. Feng, J. Wu, X. Zhu, W. Wen, X. Gong, Organ-on-a-chip: recent breakthroughs and future prospects. Biomed. Eng. Online. **19**(1), 9 (2020). 10.1186/s12938-020-0752-0 32050989 10.1186/s12938-020-0752-0 PMC7017614

[9] S.G. Kang, Y.Y. Choi, S.J. Mo, T.H. Kim, J.H. Ha, D.K. Hong, H. Lee, S.D. Park, J.-J. Shim, J.-L. Lee, B.G. Chung, Effect of gut microbiome-derived metabolites and extracellular vesicles on hepatocyte functions in a gut-liver axis chip. Nano Converg. **10**(1), 5 (2023). 10.1186/s40580-022-00350-6 36645561 10.1186/s40580-022-00350-6 PMC9842828

[10] M.-H. Kim, Y. Lee, G.M. Seo, S. Park, Advancements in Kidney-on-Chip: Antibiotic-Induced kidney Injury and future directions. BioChip J. (2024). 10.1007/s13206-024-00160-4

[11] H. Tang, Y. Abouleila, L. Si, A.M. Ortega-Prieto, C.L. Mummery, D.E. Ingber, A. Mashaghi, Human organs-on-chips for Virology. Trends Microbiol. **28**(11), 934–946 (2020). 10.1016/j.tim.2020.06.005 32674988 10.1016/j.tim.2020.06.005 PMC7357975

[12] J. Wang, C. Wang, N. Xu, Z.-F. Liu, D.-W. Pang, Z.-L. Zhang, A virus-induced kidney disease model based on organ-on-a-chip: Pathogenesis exploration of virus-related renal dysfunctions. Biomaterials. **219**, 119367 (2019). 10.1016/j.biomaterials.2019.119367 31344514 10.1016/j.biomaterials.2019.119367

[13] E. Ferrari, C. Palma, S. Vesentini, P. Occhetta, M. Rasponi, Integrating biosensors in Organs-on-Chip devices: a perspective on current strategies to monitor Microphysiological systems. Biosensors. **10**(9), 110 (2020). 10.3390/bios10090110 32872228 10.3390/bios10090110 PMC7558092

[14] Y. Zhu, K. Mandal, A.L. Hernandez, S. Kawakita, W. Huang, P. Bandaru, S. Ahadian, H.-J. Kim, V. Jucaud, M.R. Dokmeci, A. Khademhosseini, State of the art in integrated biosensors for organ-on-a-chip applications. Curr. Opin. Biomed. Eng. **19**, 100309 (2021). 10.1016/j.cobme.2021.100309 37206309 10.1016/j.cobme.2021.100309 PMC10193909

[15] A. Kaushik Fics Mrcs, E. Acs, A. Ruiz, S. Bhansali, M. Nair, Miniaturized sensing devices for Biomarker Detection. J. Biosens. Bioelectron. **5**, 1000e132 (2014). 10.4172/2155-6210.1000e132

[16] V.J. Costela-Ruiz, R. Illescas-Montes, J.M. Puerta-Puerta, C. Ruiz, L. Melguizo-Rodríguez, SARS-CoV-2 infection: the role of cytokines in COVID-19 disease. Cytokine Growth Factor. Rev. **54**, 62–75 (2020). 10.1016/j.cytogfr.2020.06.001 32513566 10.1016/j.cytogfr.2020.06.001 PMC7265853

[17] R.-J. Hsu, W.-C. Yu, G.-R. Peng, C.-H. Ye, S. Hu, P.C.T. Chong, K.Y. Yap, J.Y.C. Lee, W.-C. Lin, S.-H. Yu, The role of cytokines and chemokines in severe Acute Respiratory Syndrome Coronavirus 2 infections. Front. Immunol. 13 (2022). 10.3389/fimmu.2022.832394 10.3389/fimmu.2022.832394 PMC9021400 35464491

[18] S.A. Jones, Directing transition from Innate to Acquired Immunity: defining a role for IL-61. J. Immun. **175**(6), 3463–3468 (2005). 10.4049/jimmunol.175.6.3463 16148087 10.4049/jimmunol.175.6.3463

[19] M. KOPF, F. A. RAMSAY, H. BROMBACHER, G. BAUMANN, C. FREER, J.-C. GALANOS, G. GUTIERREZ-RAMOS, KÖHLER, Pleiotropic defects of IL-6—deficient mice including early hematopoiesis, T and B cell function, and Acute phase responses. Ann. N Y Acad. Sci. **762**(1), 308–318 (1995). 10.1111/j.1749-6632.1995.tb32335.x 10.1111/j.1749-6632.1995.tb32335.x 7545368

[20] E.A. Said, I. Al-Reesi, N. Al-Shizawi, S. Jaju, M.S. Al-Balushi, C.Y. Koh, A.A. Al-Jabri, L. Jeyaseelan, Defining IL-6 levels in healthy individuals: a meta-analysis. J. Med. Virol. **93**(6), 3915–3924 (2021). 10.1002/jmv.26654 33155686 10.1002/jmv.26654

[21] Y. Nazerian, M. Ghasemi, Y. Yassaghi, A. Nazerian, S.M. Hashemi, Role of SARS-CoV-2-induced cytokine storm in multi-organ failure: molecular pathways and potential therapeutic options. Int. Immunopharmacol. **113**, 109428 (2022). 10.1016/j.intimp.2022.109428 36379152 10.1016/j.intimp.2022.109428 PMC9637536

[22] L. Li, J. Li, M. Gao, H. Fan, Y. Wang, X. Xu, C. Chen, J. Liu, J. Kim, R. Aliyari, J. Zhang, Y. Jin, X. Li, F. Ma, M. Shi, G. Cheng, H. Yang, Interleukin-8 as a Biomarker for Disease Prognosis of Coronavirus Disease-2019 patients. Front. Immunol. **11** (2021). 10.3389/fimmu.2020.602395 10.3389/fimmu.2020.602395 PMC7820901 33488599

[23] T.P. Velavan, C.G. Meyer, Mild versus severe COVID-19: Laboratory markers. Int. J. Infect. Dis. **95**, 304–307 (2020). 10.1016/j.ijid.2020.04.061 10.1016/j.ijid.2020.04.061 PMC7194601 32344011

[24] M.D. Tracey, J. Kevin, P.D. Cerami, Anthony, TUMOR NECROSIS FACTOR: a pleiotropic cytokine and Therapuetic Target. Annu. Rev. Med. **45**, 491–503 (1994). 10.1146/annurev.med.45.1.491 8198398 10.1146/annurev.med.45.1.491

[25] A.A. Gharamti, O. Samara, A. Monzon, G. Montalbano, S. Scherger, K. DeSanto, D.B. Chastain, S. Sillau, J.G. Montoya, C. Franco-Paredes, A.F. Henao-Martínez, L. Shapiro, Proinflammatory cytokines levels in sepsis and healthy volunteers, and tumor necrosis factor-alpha associated sepsis mortality: a systematic review and meta-analysis. Cytokine. **158**, 156006 (2022). 10.1016/j.cyto.2022.156006 36044827 10.1016/j.cyto.2022.156006

[26] F. Villinger, P.E. Rollin, S.S. Brar, N.F. Chikkala, J. Winter, J.B. Sundstrom, S.R. Zaki, R. Swanepoel, A.A. Ansari, C.J. Peters, Markedly Elevated Levels of Interferon (IFN)-γ, IFN-α, interleukin (IL)-2, IL-10, and Tumor Necrosis Factor-α Associated with Fatal Ebola Virus infection. J. Infect. Dis. **179**, S188–S191 (1999). 10.1086/514283 9988183 10.1086/514283

[27] S.H. Seo, R.G. Webster, Tumor necrosis factor alpha exerts powerful Anti-influenza Virus effects in Lung epithelial cells. J. Virol. **76**(3), 1071–1076 (2002). 10.1128/jvi.76.3.1071-1076.2002 11773383 10.1128/JVI.76.3.1071-1076.2002 PMC135862

[28] R. Karki, B.R. Sharma, S. Tuladhar, E.P. Williams, L. Zalduondo, P. Samir, M. Zheng, B. Sundaram, B. Banoth, R.S. Malireddi, Synergism of TNF-α and IFN-γ triggers inflammatory cell death, tissue damage, and mortality in SARS-CoV-2 infection and cytokine shock syndromes. Cell. **184**(1), 149–168 (2021). 10.1016/j.cell.2020.11.025 33278357 10.1016/j.cell.2020.11.025 PMC7674074

[29] J. Palomo, D. Dietrich, P. Martin, G. Palmer, C. Gabay, The interleukin (IL)-1 cytokine family – balance between agonists and antagonists in inflammatory diseases. Cytokine. **76**(1), 25–37 (2015). 10.1016/j.cyto.2015.06.017 26185894 10.1016/j.cyto.2015.06.017

[30] A. Richardson, N. Terrazzini, C. Gage, B.J. Lee, R. Bradley, P. Watt, E.R. Watkins, Inflammatory and psychological consequences of chronic high exposure firefighting. J. Therm. Biol. **111**, 103399 (2023). 10.1016/j.jtherbio.2022.103399 36585074 10.1016/j.jtherbio.2022.103399

[31] O.J. McElvaney, N.L. McEvoy, O.F. McElvaney, T.P. Carroll, M.P. Murphy, D.M. Dunlea, O. Ní Choileáin, J. Clarke, E. O’Connor, G. Hogan, D. Ryan, I. Sulaiman, C. Gunaratnam, P. Branagan, M.E. O’Brien, R.K. Morgan, R.W. Costello, K. Hurley, S. Walsh, E. de Barra, C. McNally, S. McConkey, F. Boland, S. Galvin, F. Kiernan, J. O’Rourke, R. Dwyer, M. Power, P. Geoghegan, C. Larkin, R.A. O’Leary, J. Freeman, A. Gaffney, B. Marsh, G.F. Curley, N.G. McElvaney, Characterization of the inflammatory response to severe COVID-19 illness. Am. J. Respir Crit. Care Med. **202**(6), 812–821 (2020). 10.1164/rccm.202005-1583OC 32584597 10.1164/rccm.202005-1583OC PMC7491404

[32] S. Makaremi, A. Asgarzadeh, H. Kianfar, A. Mohammadnia, V. Asghariazar, E. Safarzadeh, The role of IL-1 family of cytokines and receptors in pathogenesis of COVID-19. Inflamm. Res. **71**(7), 923–947 (2022). 10.1007/s00011-022-01596-w 35751653 10.1007/s00011-022-01596-w PMC9243884

[33] C.-M. Chu, I.S. Sheen, C.-T. Yeh, S.-Y. Hsieh, S.-L. Tsai, Y.-F. Liaw, Serum levels of interferon-α and-γ in acute and chronic hepatitis B virus infection. Dig. Dis. Sci. **40**(10), 2107–2112 (1995). 10.1007/BF02208991 10.1007/BF02208991 7587774

[34] M. Sato, M. Hosoya, P.F. Wright, Differences in serum cytokine levels between influenza virus A and B infections in children. Cytokine. **47**(1), 65–68 (2009). 10.1016/j.cyto.2009.05.003 19497758 10.1016/j.cyto.2009.05.003

[35] T. Herold, V. Jurinovic, C. Arnreich, B.J. Lipworth, J.C. Hellmuth, M. von Bergwelt-Baildon, M. Klein, T. Weinberger, Elevated levels of IL-6 and CRP predict the need for mechanical ventilation in COVID-19. J. Allergy Clin. Immunol. **146**(1), 128–136e4 (2020). 10.1016/j.jaci.2020.05.008 10.1016/j.jaci.2020.05.008 PMC7233239 32425269

[36] F. Islam, S. Habib, K. Badruddza, M. Rahman, M.R. Islam, S. Sultana, A. Nessa, The Association of Cytokines IL-2, IL-6, TNF-α, IFN-γ, and IL-10 with the Disease Severity of COVID-19: a study from Bangladesh. Cureus. **16**(4), e57610 (2024). 10.7759/cureus.57610 38707035 10.7759/cureus.57610 PMC11069400

[37] J. Kim, J. Jeon, H. Jang, Y. Moon, A.T. Abafogi, D. van Noort, J. Lee, T. Kang, S. Park, 3D printed fluidic swab for COVID-19 testing with improved diagnostic yield and user comfort. Nano Converg. **10**(1), 45 (2023). 10.1186/s40580-023-00393-3 37715925 10.1186/s40580-023-00393-3 PMC10505115

[38] Y. Liu, Y. Li, Y. Hang, L. Wang, J. Wang, N. Bao, Y. Kim, H.W. Jang, Rapid assays of SARS-CoV-2 virus and noble biosensors by nanomaterials. Nano Converg. **11**(1), 2 (2024). 10.1186/s40580-023-00408-z 38190075 10.1186/s40580-023-00408-z PMC10774473

[39] H. Melbye, D. Hvidsten, A. Holm, S.A. Nordbø, J. Brox, The course of C-reactive protein response in untreated upper respiratory tract infection. Br. J. Gen. Pract. **54**(506), 653–658 (2004) 15353049 PMC1326064

[40] A.J. Abdullah, A.T. Arif, H.A. Rahman, K.Q. Sofihussein, J.M. Hadi, J.M.A. Aziz, S.S. Tofiq, A.M. Mustafa, Assessing serum C-reactive protein as a predictor of COVID-19 outcomes: a retrospective cross-sectional study. Ann. Med. Surg. **85**(7), 3359–3363 (2023). 10.1097/ms9.0000000000000761 10.1097/MS9.0000000000000761 PMC10328585 37427205

[41] K.F. Kernan, J.A. Carcillo, Hyperferritinemia and inflammation. Int. Immunol. **29**(9), 401–409 (2017). 10.1093/intimm/dxx031 10.1093/intimm/dxx031 PMC5890889 28541437

[42] H.A. Jackson, K. Carter, C. Darke, M.G. Guttridge, D. Ravine, R.D. Hutton, J.A. Napier, M. Worwood, HFE mutations, iron deficiency and overload in 10 500 blood donors. Br. J. Haematol. **114**(2), 474–484 (2001). 10.1046/j.1365-2141.2001.02949.x 10.1046/j.1365-2141.2001.02949.x 11529872

[43] H.H. Abed, A.G. Al-Ziaydi, I.A. Taher, A.K. Al Dulaimi, Comparison of some hematological parameters between male and female patients infected with COVID-19. Hum. Antibodies. **30**(3), 151–155 (2022). 10.3233/HAB-220006 10.3233/HAB-220006 35786649

[44] A. Marc, M. Kerioui, F. Blanquart, J. Bertrand, O. Mitjà, M. Corbacho-Monné, M. Marks, J. Guedj, Quantifying the relationship between SARS-CoV-2 viral load and infectiousness. eLife 10, e69302 (2021). 10.7554/eLife.69302 10.7554/eLife.69302 PMC8476126 34569939

[45] M.C. Luca, I.I. Loghin, I.F. Mihai, R. Popa, A. Vâţă, C. Manciuc, Liver damage Associated with SARS-CoV-2 infection—myth or reality? J. Pers. Med. **13**(2), 349 (2023). 10.3390/jpm13020349 36836583 10.3390/jpm13020349 PMC9965594

[46] H. Akbar, H. Fallatah, Serum ALT levels in a cohort of healthy blood donors and volunteers from Saudi Arabia: the influence of sex and body Mass Index. Ann. Gastroenterol. Hepatol. **1**, 13–19 (2010)

[47] R.K. Saini, N. Saini, S. Ram, S.L. Soni, V. Suri, P. Malhotra, J. Kaur, I. Verma, S. Sharma, D. Zohmangaihi, COVID-19 associated variations in liver function parameters: a retrospective study. Postgrad. Med. J. **98**(1156), 91–97 (2020). 10.1136/postgradmedj-2020-138930 33184141 10.1136/postgradmedj-2020-138930

[48] E. Bogin, G. Ziv, J. Avidar, B. Rivetz, S. Gordin, A. Saran, Distribution of lactate dehydrogenase isoenzymes in normal and inflamed bovine udders and milk. Res. Vet. Sci. **22**(2), 198–200 (1977). 10.1016/S0034-5288(18)33286-7 870959

[49] M.-. Wu, L. Yao, Y. Wang, X.-. Zhu, X.-. Wang, P.-. Tang, C. Chen, Clinical evaluation of potential usefulness of serum lactate dehydrogenase (LDH) in 2019 novel coronavirus (COVID-19) pneumonia. Respir Res. **21**(1), 171 (2020). 10.1186/s12931-020-01427-8 32631317 10.1186/s12931-020-01427-8 PMC7336103

[50] O. Saidani, M. Umer, N. Alturki, A. Alshardan, M. Kiran, S. Alsubai, T.-H. Kim, I. Ashraf, White blood cells classification using multi-fold pre-processing and optimized CNN model. Sci. Rep. **14**(1), 3570 (2024). 10.1038/s41598-024-52880-0 38347011 10.1038/s41598-024-52880-0 PMC10861568

[51] C. Qin, L. Zhou, Z. Hu, S. Zhang, S. Yang, Y. Tao, C. Xie, K. Ma, K. Shang, W. Wang, D.-S. Tian, Dysregulation of Immune response in patients with coronavirus 2019 (COVID-19) in Wuhan, China. Clin. Infect. Dis. **71**(15), 762–768 (2020). 10.1093/cid/ciaa248 10.1093/cid/ciaa248 PMC7108125 32161940

[52] S.H. Ryu, S.W. Min, J.H. Kim, H.J. Jeong, G.C. Kim, D.K. Kim, Y.-J. Sim, Diagnostic significance of fibrin degradation products and D-Dimer in patients with breast Cancer-related Lymphedema. Ann. Rehabil Med. **43**(1), 81–86 (2019). 10.5535/arm.2019.43.1.81 30852874 10.5535/arm.2019.43.1.81 PMC6409666

[53] H.-H. Yu, C. Qin, M. Chen, W. Wang, D.-S. Tian, D-dimer level is associated with the severity of COVID-19. Thromb. Res. **195**, 219–225 (2020). 10.1016/j.thromres.2020.07.047 10.1016/j.thromres.2020.07.047 PMC7384402 32777639

[54] N. Tang, D. Li, X. Wang, Z. Sun, Abnormal coagulation parameters are associated with poor prognosis in patients with novel coronavirus pneumonia. J. Thromb. Haemost. **18**(4), 844–847 (2020). 10.1111/jth.14768 32073213 10.1111/jth.14768 PMC7166509

[55] A.G. Abril, J. Alejandre, A. Mariscal, L. Alserawan, N. Rabella, E. Roman, J. Lopez-Contreras, F. Navarro, E. Serrano, J.F. Nomdedeu, S. Vidal, Titers of IgG and IgA against SARS-CoV-2 proteins and their association with symptoms in mild COVID-19 infection. Sci. Rep. **14**(1), 12725 (2024). 10.1038/s41598-024-59634-y 38830902 10.1038/s41598-024-59634-y PMC11148197

[56] H. Hou, T. Wang, B. Zhang, Y. Luo, L. Mao, F. Wang, S. Wu, Z. Sun, Detection of IgM and IgG antibodies in patients with coronavirus disease 2019. Clin. Transl Immunol. **9**(5), e1136 (2020). 10.1002/cti2.1136 10.1002/cti2.1136 PMC7202656 32382418

[57] F. Ozturk Kirbay, D. Odaci, Electrospun Poly-ε-caprolactone/Poly-l-lysine (PCL/PLL) nanofibers as an Emergent Material for the Preparation of Electrochemical Immunosensor to detect serum amyloid A. ACS Appl. Polym. Mater. **6**(7), 3778–3786 (2024). 10.1021/acsapm.3c03002

[58] C. Quan, L. Quan, Q. Wen, M. Yang, T. Li, Alanine aminotransferase electrochemical sensor based on graphene@MXene composite nanomaterials. Microchim Acta. **191**(1), 45 (2023). 10.1007/s00604-023-06131-0 10.1007/s00604-023-06131-0 38114837

[59] O. Calvo-Lozano, M. Sierra, M. Soler, M.C. Estévez, L. Chiscano-Camón, A. Ruiz-Sanmartin, J.C. Ruiz-Rodriguez, R. Ferrer, J.J. González-López, J. Esperalba, C. Fernández-Naval, L. Bueno, R. López-Aladid, A. Torres, L. Fernández-Barat, S. Attoumani, R. Charrel, B. Coutard, L.M. Lechuga, Label-free Plasmonic Biosensor for Rapid, quantitative, and highly sensitive COVID-19 serology: implementation and clinical validation. Anal. Chem. **94**(2), 975–984 (2022). 10.1021/acs.analchem.1c03850 34971311 10.1021/acs.analchem.1c03850 PMC8751014

[60] P. Thawany, A. Khanna, U.K. Tiwari, A. Deep, L-cysteine/MoS2 modified robust surface plasmon resonance optical fiber sensor for sensing of Ferritin and IgG. Sci. Rep. **13**(1), 5297 (2023). 10.1038/s41598-023-31152-3 37002282 10.1038/s41598-023-31152-3 PMC10064954

[61] N. Atar, M.L. Yola, A novel QCM immunosensor development based on gold nanoparticles functionalized sulfur-doped graphene quantum dot and h-ZnS-CdS NC for Interleukin-6 detection. Anal. Chim. Acta. **1148**, 338202 (2021). 10.1016/j.aca.2021.338202 33516376 10.1016/j.aca.2021.338202

[62] M. Pohanka, Immunoassay of interferon gamma by quartz crystal microbalance biosensor. Talanta. **218**, 121167 (2020). 10.1016/j.talanta.2020.121167 32797920 10.1016/j.talanta.2020.121167

[63] H.J. Lim, T. Saha, B.T. Tey, W.S. Tan, C.W. Ooi, Quartz crystal microbalance-based biosensors as rapid diagnostic devices for infectious diseases. Biosens. Bioelectron. **168**, 112513 (2020). 10.1016/j.bios.2020.112513 32889395 10.1016/j.bios.2020.112513 PMC7443316

[64] S. Wankar, N.W. Turner, R.J. Krupadam, Polythiophene nanofilms for sensitive fluorescence detection of viruses in drinking water. Biosens. Bioelectron. **82**, 20–25 (2016). 10.1016/j.bios.2016.03.020 27031187 10.1016/j.bios.2016.03.020

[65] L. La Spada, L. Vegni, Electromagnetic nanoparticles for sensing and medical diagnostic applications. Materials. **11**(4), 603 (2018). 10.3390/ma11040603 29652853 10.3390/ma11040603 PMC5951487

[66] J. Yoon, B.M. Conley, M. Shin, J.-H. Choi, C.K. Bektas, J.-W. Choi, K.-B. Lee, Ultrasensitive Electrochemical detection of mutated viral RNAs with single-nucleotide resolution using a nanoporous electrode array (NPEA). ACS Nano. **16**(4), 5764–5777 (2022). 10.1021/acsnano.1c10824 35362957 10.1021/acsnano.1c10824

[67] A. Łoczechin, K. Séron, A. Barras, E. Giovanelli, S. Belouzard, Y.-T. Chen, N. Metzler-Nolte, R. Boukherroub, J. Dubuisson, S. Szunerits, Functional carbon quantum dots as medical countermeasures to human coronavirus. ACS Appl. Mater. Interfaces. **11**(46), 42964–42974 (2019). 10.1021/acsami.9b15032 31633330 10.1021/acsami.9b15032 PMC7075527

[68] D. Sumbria, E. Berber, M. Mathayan, B.T. Rouse, Virus infections and host metabolism—can we manage the interactions? Front. Immunol. **11**, 594963 (2021). 10.3389/fimmu.2020.594963 33613518 10.3389/fimmu.2020.594963 PMC7887310

[69] Y. Pang, Z. Rong, J. Wang, R. Xiao, S. Wang, A fluorescent aptasensor for H5N1 influenza virus detection based-on the core–shell nanoparticles metal-enhanced fluorescence (MEF). Biosens. Bioelectron. **66**, 527–532 (2015). 10.1016/j.bios.2014.10.052 10.1016/j.bios.2014.10.052 25506900

[70] N.V. Zaytseva, R.A. Montagna, A.J. Baeumner, Microfluidic biosensor for the serotype-specific detection of dengue virus RNA. Anal. Chem. **77**(23), 7520–7527 (2005). 10.1021/ac0509206 16316157 10.1021/ac0509206

[71] C.-H. Lu, Y. Zhang, S.-F. Tang, Z.-B. Fang, H.-H. Yang, X. Chen, G.-N. Chen, Sensing HIV related protein using epitope imprinted hydrophilic polymer coated quartz crystal microbalance. Biosens. Bioelectron. **31**(1), 439–444 (2012). 10.1016/j.bios.2011.11.008 22143073 10.1016/j.bios.2011.11.008

[72] S.-H. Chen, Y.-C. Chuang, Y.-C. Lu, H.-C. Lin, Y.-L. Yang, C.-S. Lin, A method of layer-by-layer gold nanoparticle hybridization in a quartz crystal microbalance DNA sensing system used to detect dengue virus. Nanotechnology. **20**(21), 215501 (2009). 10.1088/0957-4484/20/21/215501 19423930 10.1088/0957-4484/20/21/215501

[73] E. Souza, G. Nascimento, N. Santana, D. Ferreira, M. Lima, E. Natividade, D. Martins, J. Lima-Filho, Label-free electrochemical detection of the specific oligonucleotide sequence of dengue virus type 1 on pencil graphite electrodes. Sensors. **11**(6), 5616–5629 (2011). 10.3390/s110605616 22163916 10.3390/s110605616 PMC3231433

[74] L.F. de Lima, P.P. Barbosa, C.L. Simeoni, R.F.O. de Paula, J.L. Proenca-Modena, W.R. de Araujo, Electrochemical Paper-based Nanobiosensor for Rapid and Sensitive Detection of Monkeypox Virus. ACS Appl. Mater. Interfaces. **15**(50), 58079–58091 (2023). 10.1021/acsami.3c10730 38063784 10.1021/acsami.3c10730

[75] Y. Zhang, B. Zheng, C. Zhu, X. Zhang, C. Tan, H. Li, B. Chen, J. Yang, J. Chen, Y. Huang, Single-layer transition metal dichalcogenide nanosheet-based nanosensors for rapid, sensitive, and multiplexed detection of DNA. Adv. Mater. **27**(5), 935–939 (2014). 10.1002/adma.201404568 25504749 10.1002/adma.201404568

[76] L. Chen, Z. Sheng, A. Zhang, X. Guo, J. Li, H. Han, M. Jin, Quantum-dots‐based fluoroimmunoassay for the rapid and sensitive detection of avian influenza virus subtype H5N1. Luminescence. **25**(6), 419–423 (2010). 10.1002/bio.1167 19844980 10.1002/bio.1167

[77] M.-K. Tsang, W. Ye, G. Wang, J. Li, M. Yang, J. Hao, Ultrasensitive detection of Ebola virus oligonucleotide based on upconversion nanoprobe/nanoporous membrane system. ACS Nano. **10**(1), 598–605 (2016). 10.1021/acsnano.5b05622 26720408 10.1021/acsnano.5b05622

[78] J.R. Choi, K.W. Yong, R. Tang, Y. Gong, T. Wen, H. Yang, A. Li, Y.C. Chia, B. Pingguan-Murphy, F. Xu, Lateral flow assay based on paper–hydrogel hybrid material for sensitive point‐of‐care detection of dengue virus. Adv. Healthc. Mater. **6**(1), 1600920 (2017). 10.1002/adhm.201600920 10.1002/adhm.201600920 27860384

[79] T. Leary, R. Gutierrez, A. Muerhoff, L. Birkenmeyer, S. Desai, G. Dawson, A chemiluminescent, magnetic particle-based immunoassay for the detection of hepatitis C virus core antigen in human serum or plasma. J. Med. Virol. **78**(11), 1436–1440 (2006). 10.1002/jmv.20716 16998880 10.1002/jmv.20716

[80] Y. Chen, C. Qian, C. Liu, H. Shen, Z. Wang, J. Ping, J. Wu, H. Chen, Nucleic acid amplification free biosensors for pathogen detection. Biosens. Bioelectron. **153**, 112049 (2020). 10.1016/j.bios.2020.112049 32056663 10.1016/j.bios.2020.112049

[81] Z. Akbari jonous, J.S. Shayeh, F. Yazdian, A. Yadegari, M. Hashemi, M. Omidi, An electrochemical biosensor for prostate cancer biomarker detection using graphene oxide–gold nanostructures. Eng. Life Sci. **19**(3), 206–216 (2019). 10.1002/elsc.201800093 32625003 10.1002/elsc.201800093 PMC6999480

[82] J. Li, H. Bai, Z. Wang, B. Xu, K.N. Peters Olson, C. Liu, Y. Su, J. Hao, J. Shen, X. Xi, J. Zhen, R. Yu, Y. Sun, X. Xie, W.-. Tian, F. Yu, X. Liu, L. Zhang, D. Zhou, L. Si, Advancements in organs-on-chips technology for viral disease and anti-viral research. Organs-on-a-Chip **5**, 100030 (2023). 10.1016/j.ooc.2023.100030

[83] M.-H. Kim, J. Lee, C.T. Lim, S. Park, Potential of bioprinted intestine-on-chip models in advancing understanding of human coronavirus infections and drug screening. Int. J. Bioprinting. **10**(2), 1704 (2024). 10.36922/ijb.1704

[84] H. Bai, L. Si, A. Jiang, C. Belgur, Y. Zhai, R. Plebani, C.Y. Oh, M. Rodas, A. Patil, A. Nurani, S.E. Gilpin, R.K. Powers, G. Goyal, R. Prantil-Baun, D.E. Ingber, Mechanical control of innate immune responses against viral infection revealed in a human lung alveolus chip. Nat. Commun. **13**(1), 1928 (2022). 10.1038/s41467-022-29562-4 35396513 10.1038/s41467-022-29562-4 PMC8993817

[85] M. Zhang, P. Wang, R. Luo, Y. Wang, Z. Li, Y. Guo, Y. Yao, M. Li, T. Tao, W. Chen, J. Han, H. Liu, K. Cui, X. Zhang, Y. Zheng, J. Qin, Biomimetic Human Disease Model of SARS-CoV-2-Induced Lung Injury and Immune responses on Organ Chip System. Adv. Sci. **8**(3), 2002928 (2021). 10.1002/advs.202002928 10.1002/advs.202002928 PMC7646023 33173719

[86] T. Cao, C. Shao, X. Yu, R. Xie, C. Yang, Y. Sun, S. Yang, W. He, Y. Xu, Q. Fan, F. Ye, Biomimetic Alveolus-on-a-Chip for SARS-CoV-2 infection recapitulation. Research 2022 (2022). 10.34133/2022/9819154 10.34133/2022/9819154 PMC8841031 35224503

[87] V.V. Thacker, K. Sharma, N. Dhar, G.F. Mancini, J. Sordet-Dessimoz, J.D. McKinney, Rapid Endotheliitis and vascular damage characterize SARS‐CoV‐2 infection in a human lung‐on‐chip model. EMBO Rep. **22**(6), e52744 (2021). 10.15252/embr.202152744 33908688 10.15252/embr.202152744 PMC8183417

[88] J.D. Domizio, M.F. Gulen, F. Saidoune, V.V. Thacker, A. Yatim, K. Sharma, T. Nass, E. Guenova, M. Schaller, C. Conrad, C. Goepfert, L. de Leval, C. Garnier, S. Berezowska, A. Dubois, M. Gilliet, A. Ablasser, The cGAS–STING pathway drives type I IFN immunopathology in COVID-19. Nature. **603**(7899), 145–151 (2022). 10.1038/s41586-022-04421-w 35045565 10.1038/s41586-022-04421-w PMC8891013

[89] L. Si, H. Bai, C.Y. Oh, L. Jin, R. Prantil-Baun, D.E. Ingber, Clinically relevant Influenza Virus Evolution reconstituted in a human lung airway-on-a-Chip. Microbiol. Spectr. **9**(2), e00257–e00221 (2021). 10.1128/Spectrum.00257-21 34523991 10.1128/Spectrum.00257-21 PMC8557867

[90] L. Si, H. Bai, M. Rodas, W. Cao, C.Y. Oh, A. Jiang, R. Moller, D. Hoagland, K. Oishi, S. Horiuchi, S. Uhl, D. Blanco-Melo, R.A. Albrecht, W.-C. Liu, T. Jordan, B.E. Nilsson-Payant, I. Golynker, J. Frere, J. Logue, R. Haupt, M. McGrath, S. Weston, T. Zhang, R. Plebani, M. Soong, A. Nurani, S.M. Kim, D.Y. Zhu, K.H. Benam, G. Goyal, S.E. Gilpin, R. Prantil-Baun, S.P. Gygi, R.K. Powers, K.E. Carlson, M. Frieman, B.R. tenOever, D.E. Ingber, A human-airway-on-a-chip for the rapid identification of candidate antiviral therapeutics and prophylactics. Nat. Biomed. Eng. **5**(8), 815–829 (2021). 10.1038/s41551-021-00718-9 10.1038/s41551-021-00718-9 PMC8387338 33941899

[91] K.H. Benam, R. Villenave, C. Lucchesi, A. Varone, C. Hubeau, H.-H. Lee, S.E. Alves, M. Salmon, T.C. Ferrante, J.C. Weaver, A. Bahinski, G.A. Hamilton, D.E. Ingber, Small airway-on-a-chip enables analysis of human lung inflammation and drug responses in vitro. Nat. Methods. **13**(2), 151–157 (2016). 10.1038/nmeth.3697 26689262 10.1038/nmeth.3697

[92] J.C. Nawroth, C. Lucchesi, D. Cheng, A. Shukla, J. Ngyuen, T. Shroff, A. Varone, K. Karalis, H.-H. Lee, S. Alves, G.A. Hamilton, M. Salmon, R. Villenave, Asthma Am. J. Respir Cell. Mol. Biol. **63**(5), 591–600 (2020). 10.1165/rcmb.2020-0010MA. A Microengineered Airway Lung Chip Models Key Features of Viral-induced Exacerbation of 32706623 10.1165/rcmb.2020-0010MA

[93] D. Huh, B.D. Matthews, A. Mammoto, M. Montoya-Zavala, H.Y. Hsin, D.E. Ingber, Reconstituting organ-level lung functions on a chip. Science. **328**(5986), 1662–1668 (2010). 10.1126/science.1188302 20576885 10.1126/science.1188302 PMC8335790

[94] D. Huang, T. Liu, J. Liao, S. Maharjan, X. Xie, M. Pérez, I. Anaya, S. Wang, A. Tirado Mayer, Z. Kang, W. Kong, V.L. Mainardi, C.E. Garciamendez-Mijares, G. García Martínez, M. Moretti, W. Zhang, Z. Gu, A.M. Ghaemmaghami, Y.S. Zhang, Reversed-engineered human alveolar lung-on-a-chip model. Proc. Natl. Acad. Sci. U. S. A. 118(19), e2016146118 (2021). 10.1073/pnas.2016146118 10.1073/pnas.2016146118 PMC8126776 33941687

[95] Y.B. Kang, T.R. Sodunke, J. Lamontagne, J. Cirillo, C. Rajiv, M.J. Bouchard, M. Noh, Liver sinusoid on a chip: long-term layered co-culture of primary rat hepatocytes and endothelial cells in microfluidic platforms. Biotechnol. Bioeng. **112**(12), 2571–2582 (2015). 10.1002/bit.25659 25994312 10.1002/bit.25659

[96] Y.B. Kang, S. Rawat, N. Duchemin, M. Bouchard, M. Noh, Human liver sinusoid on a chip for Hepatitis B Virus Replication Study. Micromachines. **8**(1), 27 (2017). 10.3390/mi8010027

[97] A.M. Ortega-Prieto, J.K. Skelton, S.N. Wai, E. Large, M. Lussignol, G. Vizcay-Barrena, D. Hughes, R.A. Fleck, M. Thursz, M.T. Catanese, M. Dorner, 3D microfluidic liver cultures as a physiological preclinical tool for hepatitis B virus infection. Nat. Commun. **9**(1), 682 (2018). 10.1038/s41467-018-02969-8 29445209 10.1038/s41467-018-02969-8 PMC5813240

[98] R. Villenave, S.Q. Wales, T. Hamkins-Indik, E. Papafragkou, J.C. Weaver, T.C. Ferrante, A. Bahinski, C.A. Elkins, M. Kulka, D.E. Ingber, Human Gut-On-A-Chip supports polarized infection of coxsackie B1 Virus in Vitro. PLOS ONE. **12**(2), e0169412 (2017). 10.1371/journal.pone.0169412 28146569 10.1371/journal.pone.0169412 PMC5287454

[99] Y. Guo, R. Luo, Y. Wang, P. Deng, T. Song, M. Zhang, P. Wang, X. Zhang, K. Cui, T. Tao, Z. Li, W. Chen, Y. Zheng, J. Qin, SARS-CoV-2 induced intestinal responses with a biomimetic human gut-on-chip. Sci. Bull. **66**(8), 783–793 (2021). 10.1016/j.scib.2020.11.015 10.1016/j.scib.2020.11.015 PMC7704334 33282445

[100] A. Bein, S. Kim, G. Goyal, W. Cao, C. Fadel, A. Naziripour, S. Sharma, B. Swenor, N. LoGrande, A. Nurani, V.N. Miao, A.W. Navia, C.G.K. Ziegler, J.O. Montañes, P. Prabhala, M.S. Kim, R. Prantil-Baun, M. Rodas, A. Jiang, L. O’Sullivan, G. Tillya, A.K. Shalek, D.E. Ingber, Enteric coronavirus infection and treatment modeled with an Immunocompetent Human Intestine-On-A-Chip. Front. Pharmacol. **12**, 718484 (2021). 10.3389/fphar.2021.718484 10.3389/fphar.2021.718484 PMC8573067 34759819

[101] B.N. Johnson, K.Z. Lancaster, I.B. Hogue, F. Meng, Y.L. Kong, L.W. Enquist, McAlpine, 3D printed nervous system on a chip. Lab. Chip. **16**(8), 1393–1400 (2016). 10.1039/C5LC01270H 26669842 10.1039/c5lc01270h PMC4829438

[102] T.P. Buzhdygan, B.J. DeOre, A. Baldwin-Leclair, T.A. Bullock, H.M. McGary, J.A. Khan, R. Razmpour, J.F. Hale, P.A. Galie, R. Potula, A.M. Andrews, S.H. Ramirez, The SARS-CoV-2 spike protein alters barrier function in 2D static and 3D microfluidic in-vitro models of the human blood–brain barrier. Neurobiol. Dis. **146**, 105131 (2020). 10.1016/j.nbd.2020.105131 33053430 10.1016/j.nbd.2020.105131 PMC7547916

[103] D. Liu, M. Zhu, Y. Lin, M. Li, R. Huang, L. Yang, Y. Song, Y. Diao, C. Yang, LY6E protein facilitates adeno-associated virus crossing in a biomimetic chip model of the human blood–brain barrier. Lab. Chip. **22**(21), 4180–4190 (2022). 10.1039/D2LC00698G 36165190 10.1039/d2lc00698g

[104] N.A. Boghdeh, K.H. Risner, M.D. Barrera, C.M. Britt, D.K. Schaffer, F. Alem, J.A. Brown, J.P. Wikswo, A. Narayanan, Application of a human blood brain barrier organ-on-a-Chip model to Evaluate Small Molecule effectiveness against Venezuelan equine Encephalitis Virus. Viruses. **14**(12), 2799 (2022). 10.3390/v14122799 36560802 10.3390/v14122799 PMC9786295

[105] Y.S. Zhang, J. Aleman, S.R. Shin, T. Kilic, D. Kim, S.A. Mousavi Shaegh, S. Massa, R. Riahi, S. Chae, N. Hu, H. Avci, W. Zhang, A. Silvestri, A. Sanati Nezhad, A. Manbohi, F. De Ferrari, A. Polini, G. Calzone, N. Shaikh, P. Alerasool, E. Budina, J. Kang, N. Bhise, J. Ribas, A. Pourmand, A. Skardal, T. Shupe, C.E. Bishop, M.R. Dokmeci, A. Atala, A. Khademhosseini, Multisensor-integrated organs-on-chips platform for automated and continual in situ monitoring of organoid behaviors. Proc. Natl. Acad. Sci. U. S. A. 114(12), E2293-E2302 (2017). 10.1073/pnas.1612906114 10.1073/pnas.1612906114 PMC5373350 28265064

[106] Y.S. Zhang, A. Khademhosseini, Adv. Eng. Hydrogels Sci. **356**(6337), eaaf3627 (2017). 10.1126/science.aaf3627 10.1126/science.aaf3627 PMC5841082 28473537

[107] I.E. Tothill, Biosensors for cancer markers diagnosis. Semin. Cell. Dev. Biol. **20**(1), 55–62 (2009). 10.1016/j.semcdb.2009.01.015 10.1016/j.semcdb.2009.01.015 19429492

[108] N.M.M. Pires, T. Dong, U. Hanke, N. Hoivik, Recent developments in optical detection technologies in lab-on-a-chip devices for biosensing applications. Sensors. **14**(8), 15458–15479 (2014). 10.3390/s140815458 25196161 10.3390/s140815458 PMC4178989

[109] A.D. van der Meer, A. van den Berg, Organs-on-chips: breaking the in vitro impasse. Integr. Biol. **4**(5), 461–470 (2012). 10.1039/c2ib00176d 10.1039/c2ib00176d 22388577

[110] S. Deng, C. Li, J. Cao, Z. Cui, J. Du, Z. Fu, H. Yang, P. Chen, Organ-on-a-chip meets artificial intelligence in drug evaluation. Theranostics. **13**(13), 4526–4558 (2023). 10.7150/thno.87266 37649608 10.7150/thno.87266 PMC10465229

[111] J. Li, J. Chen, H. Bai, H. Wang, S. Hao, Y. Ding, B. Peng, J. Zhang, L. Li, W. Huang, An overview of organs-on-chips based on deep learning. Research 2022 (2022). 10.34133/2022/9869518 10.34133/2022/9869518 PMC8795883 35136860

[112] S.R. Shin, C. Zihlmann, M. Akbari, P. Assawes, L. Cheung, K. Zhang, V. Manoharan, Y.S. Zhang, M. Yüksekkaya, K.-. Wan, M. Nikkhah, M.R. Dokmeci, X. Tang, A. Khademhosseini, Reduced Graphene Oxide-GelMA Hybrid Hydrogels as scaffolds for Cardiac tissue Engineering. Small. **12**(27), 3677–3689 (2016). 10.1002/smll.201600178 27254107 10.1002/smll.201600178 PMC5201005

[113] A. Asif, K.H. Kim, F. Jabbar, S. Kim, K.H. Choi, Real-time sensors for live monitoring of disease and drug analysis in microfluidic model of proximal tubule. Microfluid. Nanofluidics. **24**(6), 43 (2020). 10.1007/s10404-020-02347-1

[114] E.M. Shaughnessey, S.H. Kann, H. Azizgolshani, L.D. Black, J.L. Charest, E.M. Vedula, Evaluation of rapid transepithelial electrical resistance (TEER) measurement as a metric of kidney toxicity in a high-throughput microfluidic culture system. Sci. Rep. **12**(1), 13182 (2022). 10.1038/s41598-022-16590-9 35915212 10.1038/s41598-022-16590-9 PMC9343646

[115] S.A. Lim, M.U. Ahmed, Electrochemical immunosensors and their recent nanomaterial-based signal amplification strategies: a review. RSC Adv. **6**(30), 24995–25014 (2016). 10.1039/C6RA00333H

[116] S.X. Leng, J.E. McElhaney, J.D. Walston, D. Xie, N.S. Fedarko, G.A. Kuchel, ELISA and Multiplex Technologies for Cytokine Measurement in inflammation and Aging Research. J. Gerontol. A **63**(8), 879–884 (2008). 10.1093/gerona/63.8.879 10.1093/gerona/63.8.879 PMC2562869 18772478

[117] M.A. Ortega, X. Fernández-Garibay, A.G. Castaño, F. De Chiara, A. Hernández-Albors, J. Balaguer-Trias, J. Ramón-Azcón, Muscle-on-a-chip with an on-site multiplexed biosensing system for in situ monitoring of secreted IL-6 and TNF-α. Lab. Chip. **19**(15), 2568–2580 (2019). 10.1039/C9LC00285E 31243422 10.1039/c9lc00285e

[118] H. Liu, O.A. Bolonduro, N. Hu, J. Ju, A.A. Rao, B.M. Duffy, Z. Huang, L.D. Black, B.P. Timko, Heart-on-a-Chip model with Integrated Extra- and Intracellular Bioelectronics for Monitoring Cardiac Electrophysiology under Acute Hypoxia. Nano Lett. **20**(4), 2585–2593 (2020). 10.1021/acs.nanolett.0c00076 32092276 10.1021/acs.nanolett.0c00076

[119] H.M.U. Farooqi, M.A.U. Khalid, K.H. Kim, S.R. Lee, K.H. Choi, Real-time physiological sensor-based liver-on-a-Chip device for monitoring drug toxicity. J. Micromech Microeng. **30**(11), 115013 (2020). 10.1088/1361-6439/ababf4

[120] S.R. Shin, Y.S. Zhang, D.-J. Kim, A. Manbohi, H. Avci, A. Silvestri, J. Aleman, N. Hu, T. Kilic, W. Keung, M. Righi, P. Assawes, H.A. Alhadrami, R.A. Li, M.R. Dokmeci, A. Khademhosseini, Cardiac Biomarkers Anal. Chem. **88**(20), 10019–10027 (2016). 10.1021/acs.analchem.6b02028. Aptamer-Based Microfluidic Electrochemical Biosensor for Monitoring Cell-Secreted Trace 27617489 10.1021/acs.analchem.6b02028 PMC5844853

[121] Y. Liu, Y. Liu, Z. Matharu, A. Rahimian, A. Revzin, Detecting multiple cell-secreted cytokines from the same aptamer-functionalized electrode. Biosens. Bioelectron. **64**, 43–50 (2015). 10.1016/j.bios.2014.08.034 25189099 10.1016/j.bios.2014.08.034

[122] Y. Rivenson, H. Wang, Z. Wei, K. de Haan, Y. Zhang, Y. Wu, H. Günaydın, J.E. Zuckerman, T. Chong, A.E. Sisk, L.M. Westbrook, W.D. Wallace, A. Ozcan, Virtual histological staining of unlabelled tissue-autofluorescence images via deep learning. Nat. Biomed. Eng. **3**(6), 466–477 (2019). 10.1038/s41551-019-0362-y 31142829 10.1038/s41551-019-0362-y

